# Binding of the GTPase Sar1 to a Lipid Membrane Monolayer: Insertion and Orientation Studied by Infrared Reflection–Absorption Spectroscopy

**DOI:** 10.3390/polym9110612

**Published:** 2017-11-14

**Authors:** Christian Schwieger, Annette Meister, Sebastian Daum, Alfred Blume, Kirsten Bacia

**Affiliations:** Institute of Chemistry, Physical Chemistry, Martin Luther University Halle-Wittenberg, 06099 Halle, Germany; annette.meister@chemie.uni-halle.de (A.M.); sebastian.daum@chemie.uni-halle.de (S.D.); alfred.blume@chemie.uni-halle.de (A.B.)

**Keywords:** Sar1, amphipathic helix, COPII, IRRAS, maximal insertion pressure, lipid monolayer

## Abstract

Membrane-interacting proteins are polyphilic polymers that engage in dynamic protein–protein and protein–lipid interactions while undergoing changes in conformation, orientation and binding interfaces. Predicting the sites of interactions between such polypeptides and phospholipid membranes is still a challenge. One example is the small eukaryotic GTPase Sar1, which functions in phospholipid bilayer remodeling and vesicle formation as part of the multimeric coat protein complex (COPII). The membrane interaction of Sar1 is strongly dependent on its N-terminal 23 amino acids. By monolayer adsorption experiments and infrared reflection-absorption spectroscopy (IRRAS), we elucidate the role of lipids in inducing the amphipathicity of this N-terminal stretch, which inserts into the monolayer as an amphipathic helix (AH). The AH inserting angle is determined and is consistent with the philicities and spatial distribution of the amino acid monomers. Using an advanced method of IRRAS data evaluation, the orientation of Sar1 with respect to the lipid layer prior to the recruitment of further COPII proteins is determined. The result indicates that only a slight reorientation of the membrane-bound Sar1 is needed to allow coat assembly. The time-course of the IRRAS analysis corroborates a role of slow GTP hydrolysis in Sar1 desorption from the membrane.

## 1. Introduction

In eukaryotic cells, the necessity to exchange lipid and protein components between different cellular compartments requires a selective remodeling of lipid membranes and the formation of transport vesicles. This process is mediated by specialized coat protein complexes with an intrinsic vesicle fission capability [1]. The coat protein complex II (COPII) assembles on the endoplasmic reticulum membrane from mostly cytosolic protein components. Sar1 initiates the coat formation by insertion of an amphipathic helix into the membrane and recruits the Sec23/24 heterodimer to form an inner protein coat on the membrane. Finally, Sec13/31 binds and promotes outer coat polymerization, membrane deformation and liberation of lipid vesicles from the donor membrane [2].

The small GTPase binds guanine nucleotides and adopts two different conformations, depending on whether GDP or GTP occupies the nucleotide binding site [3,4,5,6]. Sar1 contains a 23-amino-acid amphipathic N_-_terminus that mediates the initial steps in COPII coat assembly [7]. In addition, Sar1 plays a key role in directly generating membrane curvature due to the insertion of its N-terminal amphipathic α-helix (AH) [8].

This step requires the activation of Sar1 by GTP. Lee et al. [8] showed that GTP binding to Sar1 is associated with a rearrangement of the switch regions to accommodate the γ-phosphate of GTP. The same authors also demonstrate that the membrane insertion of the Sar1 N-terminal helix is sufficient for generating a membrane deformation. The amphipathic nature of the Sar1 N-terminal helix is likely to be important for bending membranes, as the helix may lie partially buried in one leaflet of the lipid bilayer. Selective insertion of the AH into the cytosolic leaflet of the ER membrane would act to displace lipid headgroups, and this asymmetric binding could generate curvature toward the cytosol by the bilayer couple mechanism and/or spontaneous curvature generation [9,10,11,12].

In the present study, we address structural aspects of Sar1 binding to membranes by determining the orientation of the AH within a lipid monolayer as well as the orientation of the complete protein Sar1 with respect to the monolayer model membrane using infrared reflection–absorption spectroscopy (IRRAS). The peripheral insertion of the Sar1 AH into the cytosolic leaflet is the reason why we chose lipid monolayers as an artificial model membrane. Langmuir monolayers are convenient model systems for mimicking biological lipid bilayer membranes, which can be considered as two weakly coupled monolayers [13,14,15,16]. In recent years, IRRA spectroscopy on Langmuir monolayers has become a common strategy to study lipid–peptide and lipid–protein interactions [17,18,19,20,21,22,23,24,25]. Changes in lipid packing as well as peptide or protein secondary structure and orientation can be followed by IRRAS at desired buffer conditions, ionic strength, pH, lipid composition, and lipid lateral packing density. The aim of this study is to analyze the orientation of the Sar1 AH peptide as well as of the complete GTPase Sar1 after insertion into liquid-expanded monolayers of a model lipid mixture. Furthermore, we elucidate the importance of the lipids in inducing the organization of the N-terminal 23 amino acids into an amphipathic structure and corroborate the role of GTP hydrolysis in Sar1 desorption from the lipid layer.

## 2. Materials and Methods

### 2.1. Materials

The Sar1 protein (Sar1p) from *Saccharomyces cerevisiae* (Baker’s yeast) was recombinantly expressed in *E. coli* and purified as previously described [8,26]. The 23 amino acid N-terminal helix peptide (AH) of Sar1p was synthesized by JPT Peptide Technologies GmbH (Berlin) and a 5 mM stock solution was prepared in a 1:1 (*v*/*v*) water: trifluorethanol (TFE) mixture containing in addition 8% (*v*/*v*) of trifluoroacetic acid (TFA).

A lipid mixture (“major–minor mix”, MMM) as described in [1] was prepared, which consisted of the phospholipids DOPC (1,2-dioleoyl-*sn*-glycero-3-phosphocholine, 34.4 mol %), DOPE (1,2-dioleoyl-*sn*-glycero-3-phosphoethanolamine, 14.8 mol %), DOPS (1,2-dioleoyl-*sn*-glycero-3-[phospho-l-serine], 5.4 mol %), DOPA (1,2-dioleoyl-*sn*-glycero-3-phosphate, 3.4 mol %), soy PI (l-α-phosphatidyl-inositol, 5.4 mol %), porcine brain PI4P (l-α-phosphatidylinositol-4-phosphate, 1.5 mol %), porcine brain PI(4,5)P2 (l-α-phosphatidylinositol-4,5-bisphosphate, 0.5 mol %), and CDP-DAG (1,2-dioleoyl-*sn*-glycero-3-(cytidine diphosphate), 1.3 mol %; all lipids from Avanti Polar Lipids (Alabaster, AL, USA), and 33 mol % ergosterol (Sigma, St. Louis, MO, USA). Lipids were combined in chloroform/methanol (5:1 (*v*/*v*)) at 0.26 mM total concentration. Chloroform, methanol, 2,2,2-trifluorethanol (TFE), trifluoracetic acid (TFA), 4-(2-hydroxyethyl)-1-piperazineethanesulfonic acid (HEPES), potassium acetate, magnesium chloride, and ethylenediamine-tetraacetic acid (EDTA) were purchased from Roth (Karlsruhe, Germany). Guanosine triphosphate was purchased from Sigma (St. Louis, MO, USA). For the film balance measurements, either deionized water with a resistivity of 0.058 µS cm^−1^ (TKA Wasseraufbereitungssysteme, Thermo Electron LED GmbH, Niederelbert, Germany) or deuterium oxide from Aldrich (St. Louis, MO, USA) was used.

### 2.2. Methods

#### 2.2.1. Sar1 and AH Peptide Adsorption to the Air–Water Interface

Adsorption experiments were performed in a circular homebuilt Teflon trough with a total area of 28.23 cm^2^, and a subphase volume of 8.48 mL [27]. A Sar1 stock solution (0.1 mM) and a GTP stock solution (10 mM) were injected successively with a Hamilton syringe through a Teflon jacket just above the bottom of the trough by assuring a 13 fold molar excess of GTP over Sar1. The injection volumes were chosen to yield the final Sar1 concentrations after dilution in the trough volume (subphase concentrations) given in the respective figures. The subphase was stirred with a small stir bar to assure a homogeneous distribution of the injected protein. The surface pressure was measured by the Wilhelmy method, using a filter paper as Wilhelmy plate. The temperature of the subphase containing HKM buffer (20 mM HEPES, pH 6.8, 50 mM potassium acetate, 1.2 mM magnesium chloride), was maintained at 22 ± 0.5 °C, and a Plexiglas hood covered the trough to minimize evaporation of water. If indicated, nucleotide exchange was accelerated by chelating Mg^2+^ with 0.1 M EDTA to yield a concentration of 3 μM Mg^2+^. Adsorption experiments with the Sar1 N-terminal AH peptide were performed in a similar way using a 1:100 diluted peptide stock solution (0.05 mM peptide, 0.5% (*v*/*v*) TFE, 0.08% (*v*/*v*) TFA).

#### 2.2.2. Lipid Monolayer Preparation and Sar1 Insertion

Sar1 insertion experiments into lipid monolayers were performed with a rectangular Teflon trough (KSV-NIMA, Espoo, Finland) with a total area of 80 cm^2^, and a volume of 50 mL. The trough was filled with HKM buffer (20 mM HEPES, pH 6.8, 50 mM potassium acetate, 1.2 mM magnesium chloride) and the temperature of the subphase was maintained at 22 ± 0.5 °C. A Plexiglas hood covered the trough to minimize evaporation of water. The surface pressure was measured by the Wilhelmy method. Monolayers composed of “major–minor mix”-lipids were formed by spreading the lipid solution (0.26 mM) in a mixture of chloroform and methanol (5:1 (*v*/*v*)) onto the HKM buffer subphase. After an equilibration period of at least 10 min, the lipid film was compressed up to the desired surface pressure at a constant compression speed of 2 Å molecule^−1^ min^−1^. Insertion experiments were performed by injection of a Sar1 stock solution (0.1 mM) and GTP stock solution (10 mM) into the subphase. Lipid–protein interactions were studied either at constant surface pressure or at constant surface area. If indicated, nucleotide exchange was accelerated by chelating Mg^2+^ with 0.1 M EDTA to yield a concentration of 3 μM Mg^2+^.

#### 2.2.3. IRRAS Measurements

IRRAS experiments were performed with a two-trough-system (Riegler & Kirstein, Berlin, Germany) positioned on a moveable platform to be able to shuttle between sample and reference trough [28]. Measurements were performed in the small circular trough with a total area of 28 cm^2^, and a subphase volume of 7.8 mL. The larger trough (300 × 65 × 2 mm^3^) served as reference trough. All measurements were performed on H_2_O or D_2_O subphase (as indicated), containing 20 mM HEPES, 50 mM potassium acetate, 1.2 mM magnesium chloride (HKM buffer) at pH or pD = 6.8. The temperature of the subphase was maintained at 20 ± 1 °C by means of a circulating water bath. The surface pressure was measured using a filter paper as Wilhelmy plate. A Plexiglas hood covered the whole setup to minimize evaporation of H_2_O or D_2_O. An identical height of the subphase level in both troughs was adjusted continuously by means of a laser controlled pumping system [28].

The “major–minor mix”-lipids (0.26 mM) were spread onto the HKM buffer subphase in the circular trough up to the desired surface pressure. After an equilibration period of at least 30 min insertion experiments were started by injection of a Sar1 stock solution (0.1 mM) and GTP (10 mM) or GMP-PNP (10 mM) into the subphase of the sample trough, in amounts yielding final bulk concentrations of 100 nM Sar1 and 1.6 μM GTP or 200 μM GMP-PNP. For experiments with the AH peptide, the diluted stock solution (0.05 mM peptide) was injected yielding subphase concentrations of 100 nM AH. Experiments with the AH were performed without addition of any nucleotide. Control experiments were performed to ascertain that TFE absorption does not interfere with the absorption of the AH amide I’ vibration.

Infrared spectra were recorded with a Vertex FT-IR spectrometer (Bruker, Karlsruhe, Germany) connected to an A511 reflection unit (Bruker), and an external narrow band MCT detector and the trough system described above. The IR beam is focused by several mirrors onto the water surface at different angles of incidence (AoI, *φ*). A computer controlled rotating KRS-5 polarizer was used to generate parallel (p) and perpendicularly (s) polarized light. The shuttle technique diminishes the spectral interferences due to the water vapor absorption in the light beam [29]. For measurements at different angles of incidence, it was varied between 30° and 70° with respect to the layer normal in increments of 4°. At each AoI spectra were recorded with p-polarized and s-polarized IR radiation. After injection of the peptide or protein and nucleotide, several cycles of AoI and polarization dependent spectra were measured, with one cycle needing ~3 h of data acquisition. Spectra were recorded at a spectral resolution of 8 cm^−1^ (AH and Sar1 measurements in the presence of GTP) or 4 cm^−1^ (Sar1 measurements in the presence of GMP-PNP) using Blackman-Harris-4-Term apodization and a zero filling factor of 2. For each spectrum, 1000 scans (s-polarization) or 2000 scans (p-polarization) were averaged over a total acquisition time of ≈6–9 min. The reflection absorption spectrum (*RA*) was calculated from the single-beam reflectance spectra of the reference (*R*_0_) and the monolayer (*R*), respectively, as *RA* = −lg(*R*/*R*_0_). Spectra recording and processing were performed using the OPUS software (Bruker, Germany).

#### 2.2.4. IRRAS Data Analysis

To further reduce the contribution of rotation/vibration absorption bands of the ambient water and/or D_2_O vapor in the amide I’ band region, several spectra recorded at the same AoI and polarization were averaged. Spectra being recorded within the first 7 h or 10 h after injection of the peptide or protein, respectively, were not used for data evaluation, because the system is still prone to concentration fluctuation of the protein at the interface and/or the interface is not homogeneous. A linear baseline was subtracted between 1610 cm^−1^ and 1690 cm^−1^ in case of the AH peptide spectra and between 1595 cm^−1^ and 1695 cm^−1^ in case of the Sar1 spectra.

#### 2.2.5. Simulation and Fitting of IRRA Spectra

IRRA spectra were simulated and fitted using a self-written MATLAB program. We adopted the optical model of Kuzmin and Michailov [30,31] in a way that allows simulation of IRRA spectra with multiple bands. The optical constants (wavelength dependent values of refractive index and absorption coefficient) of the H_2_O or D_2_O subphase were taken from Bertie et al. [32,33].

For the fit of the OH stretching vibrational band we used the refractive index and absorption coefficient of the H_2_O as reported by Bertie et al. [33] shifted by −15 cm^−1^_,_ which yield better and more reliable fits. The details of the simulation procedure are given in Schwieger et al. [34].

**Fitting of AH IRRA spectra.** Simulated spectra were fitted to the experimental spectra of the AH peptide in a global fit, where all spectra recorded at different AoI and different polarisation were fitted in one non-linear least squares minimization using the *trust-region-reflective* algorithm. Before fitting linear baselines were subtracted from the simulated spectra in the same manner as described above for the experimental ones. The experimental bands were fitted in the range of 1630 cm^−1^ to 1660 cm^−1^ with one single band component at 1645 cm^−1^. Spectra measured at an AoI between 50° and 60° were not included into the fit, due to low reflectivity leading to poor signal to noise ratio close to the Brewster angle. Free fitting parameters were the tilt angle *θ* the absorption coefficient *k* and the full width at half height (*fwhh*) of the band. The polarizer quality was set to *Г* = 0.007, the refractive index of the film was set to *n* = 1.41, which corresponds to the refractive index of a phospholipid monolayer [35], assuming that the contribution of the adsorbed AH to the reflective index is negligible. Prior to the amide band fit, the layer thickness was determined by a fit of the OD absorption band and set to 2 nm. The polar angle, i.e., the angle between helix axis and transition dipole moment (TDM) of the amide I’ vibration was varied in the range of 30°–42° (see Section 3).

**Fitting of Sar1 IRRA spectra.** The analysis of the amide I’ bands of the complete protein Sar1 is more complex than the analysis described above, due to the contribution of a multitude of secondary structure elements with different orientation and absorption properties to the overall amide I’ band contour. The analysis includes fitting of the IRRA spectra at a complete set of possible Sar1 orientations, being defined by protein tilt and twist angles (Supplementary Materials, Figure S4), as well as several steps of predefining simulation parameters. The overall amide band is defined by 24 components (Supplementary Materials, Table S2), as deduced from the X-ray crystal structure 1M2O [2]. All protein orientations are given with respect to the coordinates of this crystal structure.

The **layer thickness** (*d* = 2.33 nm) and the **refractive index** of the interfacial film (*n* = 1.407) were determined from the fit of the OH stretching vibrational band, measured in an independent experiment on an H_2_O based subphase (Supplementary Materials, Figure S3).

Initial **band positions** (*υ*_0_) for the contribution of helices, β-sheets, unordered stretches, turns and an additional high wavenumber component for antiparallel β-sheets were determined from a second derivative ATR-IR measurement of a Sar1 solution (Supplementary Materials, Figure S1). These band positions were further refined by fitting the recorded IRRA spectra in the amide I’ region at with a monolayer model, including 5 spectral components (Supplementary Materials, Figure S2). In this fit, the wavenumbers determined by ATR were allowed to vary by ±5 cm^−1^. The determined band positions *υ*_0(1–5)_ (Supplementary Materials, Table S1) are comparable with values reported in Literature [36,37]. From the same fit the bands widths (full width at half hight, *fwhh*) were determined. The intensity of the subcomponents (*fwhh*_i_·*k_i_*) was set proportional to the number of amino acids contained in the respective type of secondary structure. The absorption coefficient of all β-sheet structure was further multiplied with a factor of ***k*_sheet_**/***k*_helix_** = 1.36, as for the same number of amino acids β-sheets absorb more IR light than helices and random structures [38]. The high wavenumber component of antiparallel sheets was assumed to absorb with a strength of ***k*_anti_**/***k*_helix_** = 0.19. All other secondary structure elements were assumed to have the same absorption strength.

The length and orientation of the secondary structure elements was determined from the Sar1 crystal structure as given by pdb 1M2O. Each helix is described by a helix main axis. The direction of this vector is defined as the direction of the largest variance of the spatial coordinates of all Cα atoms contained in the helix (eigenvector of the covariance matrix with the largest eigenvalue). Each β-sheet is described by a main axis and a minor axis. The main axis has the direction Cα_i_ → Cα_i+2_, the minor axis the direction of the carbonyl bond (C=O). The polar angle *α* is the angle between the main axis and the respective transition dipole moment (TDM). It is set to 38° for all types of helices (α and 3_10_) and to 90° for β-sheets. The azimuth angle β, i.e., the angle with respect to the sheet minor axis, is set to 0°. For the high frequency component of the β-sheet absorption band following angles were used: *α* = 0° and β = 90°.

With this set of parameters and contributions, the amide I’ bands of Sar1 can be calculated at all examined AoI and polarizations. For this calculation the contributions of all subcomponents to the real (*n*^x,y^ and *n*^z^) and imaginary (*k*^x,y^ and *k*^z^) part of the non-isotropic complex refractive index were calculated and added before calculating the reflectivity of the interfacial film (*R*) using a modified Fresnel equation. This simulated set of spectra was then fitted to the experimental ones with the 5 wavenumbers *υ*_0(1–5)_ for the 5 different types of secondary structure elements and the total absorption coefficient ***k*_tot_** being fitting parameters. As start values for *υ*_0(1–5)_, the previously determined values (see above) were used and the wavenumbers were allowed to vary by ±5 cm^−1^. ***k*****_tot_** is a measure for the concentration of adsorbed protein at the interface and can take any positive value. The goodness of the fit was evaluated by calculating the **sum square deviations** (*SSD*) between experimental and simulated spectra, according to:

(1) $$SSD_{\theta ,\psi }=\overset{\;}{\underset{\nu ,\varphi ,pol}{\sum }}{\left(RA_{exp}-RA_{sim}\right)}^{2}$$

The spectra were fitted in the range of *υ* = 1615–1680 cm^−1^ and at angles of incidence, *φ* = 30–70° in increments of 4°, where *φ* = 50°, 54°, and 58° were omitted from the fit, because of low reflectivity in the range of the Brewster angle.

This analysis was repeated for every possible protein orientation, which is defined **by a protein tilt angle *θ*** (rotation about the positive *y*-axis) and a **protein twist angle *ψ*** (rotation about the positive *z*-axis), where first the twist and then the tilt rotation has to be performed. The rotation is performed by multiplication of the original coordinates (as given in pdb 1M2O) with 2 rotation matrices:

(2) $${\left(\begin{array}{c} x\\ y\\ z \end{array}\right)}_{\theta ,\psi }=\left(\begin{array}{c} \;\cos\left(\theta \right)\\ 0\\ -\sin\left(\theta \right) \end{array}\begin{array}{c} \;0\\ \;1\\ \;0 \end{array}\begin{array}{c} \;\sin\left(\theta \right)\\ \;0\\ \;\cos\left(\theta \right) \end{array}\right)\cdot \left(\begin{array}{c} \cos\left(\psi \right)\\ \sin\left(\psi \right)\\ 0 \end{array}\begin{array}{c} \;-\sin\left(\psi \right)\\ \;\cos\left(\psi \right)\\ \;0 \end{array}\begin{array}{c} \;0\\ \;0\\ \;1 \end{array}\right)\cdot {\left(\begin{array}{c} x\\ y\\ z \end{array}\right)}_{pdb}$$

The rotation of the protein has influence on the direction of the helix and sheet main axes as well as on the sheet minor axes. All other optical and geometrical parameters and start values for the fitting were kept constant.

The determined $SSD_{\theta ,\psi }$ values are presented as a function of protein tilt *θ* and protein twist *ψ* in color coded 2D maps, where the minimal *SSD* values are presented in blue and the maximal *SSD* values in red. The protein orientation where the *SSD* becomes minimal, i.e., the orientation where the best spectral fit is achieved with the given parameters, is assumed to be the most likely orientation of Sar1 in the experiment.

Assuming independent, identically normally distributed residuals, we derive confidence regions for the protein orientation using the *SSD* values relative to the minimum of the calculated *SSD* landscape, according to:

(3) $$X=\frac{SSD-SSD_{min}}{SSD_{min}}\times \frac{n_{2}}{n_{1}}$$

This statistic follows an F-distribution with degrees of freedom *n*_1_ = 2 (as two parameters are kept constant (*θ*, *ψ*), whereas the other free parameters are fitted at every point of the hypersurface) and *n*_2_ = *N*–*p*, where *N* is the number of data points included in the fit and *p = 8* is the number of fitted parameters [39]. The cutoff-value for 95% confidence is approximately *X* = 3.

## 3. Results and Discussion

**Figure 1:**
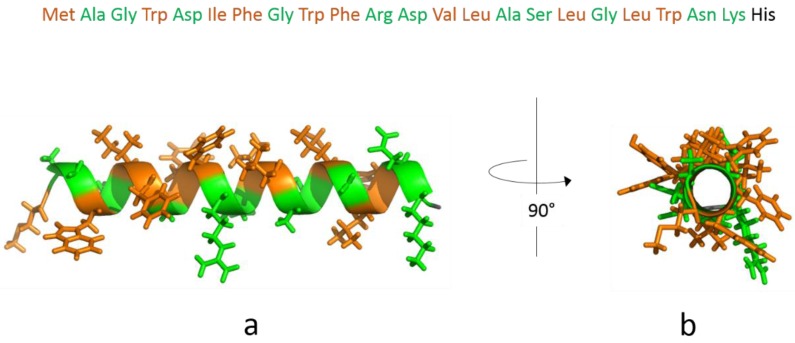
Amino acid sequence (top) and α-helical structure of the N-terminal amphipathic helix (AH) of Sar1: (**a**) side view from N-terminus (left) to C-terminus (right); and (**b**) view along the helix axis with the N-terminus in front. The colors code the amphipathicity according to a water/octanol partitioning scale [42,43] (orange: hydrophobic, green: hydrophilic). Side chains are shown only for hydrophobic (Ile, Leu, Phe, Met, Tyr, Trp, and Val) or hydrophilic (Arg, Lys, Glu, Asp, Asn, Gln, Ala, Gly, Ser, and Thr) amino acids.

The structure of the *Saccharomyces cerevisiae* Sec23/24-Sar1 pre-budding complex from the COPII vesicle coat was solved by Bi et al. [2]. The authors revealed three determinants of membrane interaction for Sec23/24-Sar1: the bow-tie shape of the complex, the positively charged concave inner surface, and the orientation of the Sar1 N-terminal AH peptide within the complex. In the GDP-bound state, the N-terminus of Sar1 is retracted into a surface pocket formed in part by the linker region of the β2-β3 hairpin [40]. In contrast, GTP binding causes a displacement of the hairpin, involving a change in register of the hydrogen bonding between strands β3 and β1, and an elimination of the pocket for the N-terminus [2]. Consequently, N-terminal residues of Sar1 become exposed and are helical structure of the AH of Sar1 (bottom). The color coding illustrates the amphipathicity able to embed into the membrane as an AH. Figure 1 shows the amino acid sequence (top) and the α-of the helix [41], with the hydrophobic face pointing up and the hydrophilic face pointing downwards.

**Figure 2:**
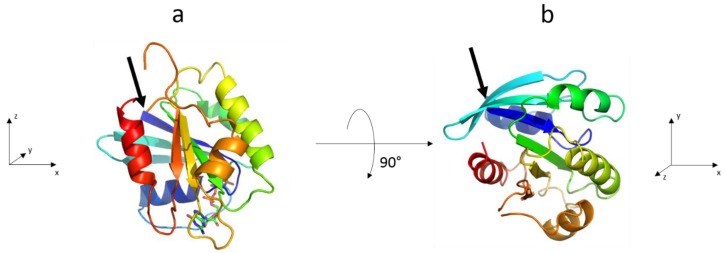
Structure of Sar1 as given in the pdb-file 1M2O, chain B [2]. The protein is shown within the pdb files coordinate system, which is depicted at the bottom of the figures and in two different representations: (**a**) looking along the positive *y*-axis; and (**b**) looking along the negative *z*-axis. The arrows indicate the N-terminus of the protein (Gly24), were the N-terminal amphipathic helix (AH) is attached. The AH is not part of the crystal structure and not shown in the figure.

The crystal structure of Sar1-GTP without N-terminal AH from Bi et al. (chain B of pdb-file 1M2O) is shown in Figure 2 in two representations. The crystallographic analysis reveals 187 amino acids (aa) whereof 90 aa are belong to helices, 37 aa to sheets, 32 aa to β turns and 33 aa to random coils. The knowledge of the number of amino acids within the secondary structure elements of Sar1 together with their relative orientation to each other was the basis for the determination of the Sar1 orientation relative to one lipid bilayer leaflet. In the following, we discuss binding and orientational changes of: (i) the Sar1 AH peptide; and (ii) the complete Sar1 protein during the insertion into an ER mimicking membrane leaflet using the Langmuir monolayer technique combined with IRRAS.

### 3.1. The Isolated Amphipathic Helix

#### 3.1.1. Monolayer Adsorption Experiments

**Figure 3:**
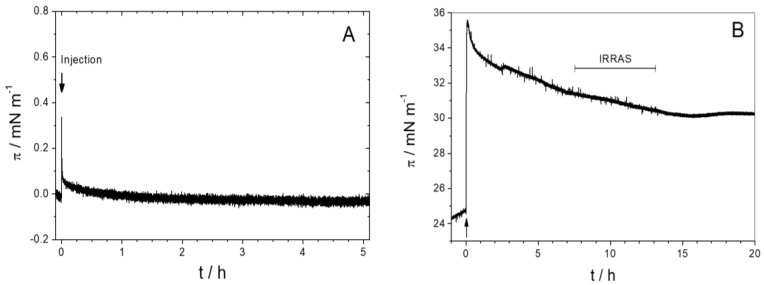
(**A**) Surface pressure versus time for the adsorption of the Sar1 23 mer N-terminal helix peptide to the air–buffer interface at a 100 nM peptide concentration starting with the injection of the appropriate volume of the stock solution into the subphase; (**B**) Injection of the AH peptide underneath an lipid monolayer (major-minor-mix, MMM) at t = 0 h. The peptide subphase concentration after injection is 100 nM. The time span, where Infrared-Refection-Absorption-Spectra (IRRAS) were detected is indicated in the figure.

We first studied the adsorption of the isolated AH peptide to the air–water interface as a function of peptide concentration. Up to a bulk concentration of 100 nM (Figure 3A), no change in surface pressure was observed upon peptide injection, indicating that the peptide has no surface activity. This observation is surprising since amphipathic α-helical peptides usually show high surface activities, especially in subphases of high ionic strength [18,44]. However, it is unclear if, after injection into the subphase, the AH peptide readily adopts an α-helical structure or whether it is in a random conformation. Circular dichroism measurements suggest a mixture of both. As the helical structure is a prerequisite for the amphipathicity, an unfolded or only partially folded state of the isolated N-terminal peptide might explain its low surface activity at the air/water interface.

**Figure 4:**
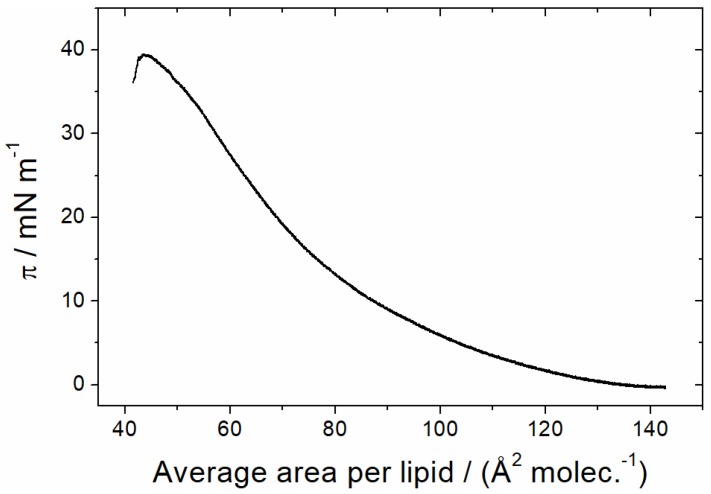
Compression isotherm of an MMM monolayer, spread on H_2_O/HKM buffer at 20 °C.

In contrast, the presence of a lipid monolayer at the interface induces the spontaneous insertion of the AH peptide into the lipid monolayer. To study the ability of AH to insert into a lipid monolayer, a lipid film was first prepared at the air–water interface of a Langmuir trough. A lipid mixture (MMM) containing zwitterionic (DOPC and DOPE) and negatively charged phospholipids (DOPS, DOPA, PI, PI(4)P, PI(4,5)P2, and CDP-DAG) as well as ergosterol, was used to mimic the lipid composition of the endoplasmic reticulum [1]. The π/A compression isotherm recorded at the air–buffer interface at 22 °C shows a liquid-expanded phase up to the monolayer collapse at 40 mN/m (Figure 4).

For the peptide insertion experiments, the lipid monolayer was prepared by spreading the lipid solution in a mixture of chloroform and methanol onto the HKM buffer subphase up to the desired surface pressure (~25 mN/m). After injection of the peptide underneath the lipid monolayer, we observe an immediate increase in surface pressure from 25 mN/m to 35 mN/m (Figure 3B). Within several hours, the monolayer then slowly relaxes to 30 mN/m, which might be due to conformational changes within the peptide, a rearrangement of the AHs within the lipid monolayer, or an instability of the monolayer. The net charge of the synthetic AH peptide is positive (+1 or +2, depending on the His protonation state and including the positive charge at the unprotected N-terminus). Electrostatic interaction between the negatively charged monolayer and peptide might therefore increase apparent binding [45]. Hydrophobic interactions between the hydrophobic side of the amphipathic helix and the lipid chains contribute to a stable insertion of the AH peptide into the membrane leaflet [12]. The peptide has a large number of hydrophobic residues as illustrated in Figure 1. The formation of an α-helix at the interface leads to the congregation of hydrophilic residues at one face of the helix and of hydrophobic residues at the opposite face of the helix. On the one hand, this allows favorable interactions of hydrophobic and hydrophilic residues with the hydrophobic and the interfacial region of the monolayer, respectively. On the other hand, the helix formation reduces the free energy cost of the transfer of the peptide from aqueous bulk phase into the monolayer [42,46]. According to the hydrophobicity scales published by the White lab [41,46] the free energy of transfer of the AH peptide to a lipid interface is negative (−16 kJ/mol), i.e., favorable, whereas the free energy of transfer of the complete AH peptide from aqueous phase into octanol is positive (+6 kJ/mol), i.e., unfavorable. This is probably due to the large number of residues that favor the membrane interface, such as tryptophans [43]. We, therefore, assume that the AH peptide localizes at the hydrophilic/hydrophobic interface of the lipid monolayer.

#### 3.1.2. IRRAS

The orientation of the adsorbed AH peptide can be determined by angle and orientation dependent IRRA spectroscopy. This is possible because intensity and sign of the amide I band depends on the angle of incidence (AoI) and on the polarization of the incoming IR light as well as on the orientation of the α-helix [17,19,23,47,48]. Thus, the tilt angle *θ* of the AH peptide can be determined when AoI and polarization are varied in a controlled manner. Most studies so far used this relationship qualitatively or relied on the analysis of a limited range of AoI (i.e., below the Brewster angle). Here, we present for the first time a quantitative study using a wide range of AoI (i.e., below and above the Brewster angle) to determine the insertion angle of the AH peptide.

**Figure 5:**
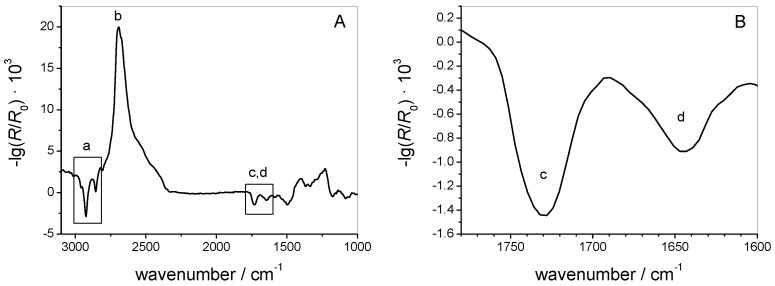
(**A**) IRRA spectra detected in p-polarization at an AoI of 40° of an MMM monolayer after adsorption of AH: (a) CH_2_ stretching vibration of the lipid acyl chains; (b) D_2_O stretching vibrations due to subphase absorption; (c) carbonyl stretching vibration of the lipid head groups; and (d) amide I’ vibration of the adsorbed peptide. (**B**) Close-up view of the region of: carbonyl (c); and amide I’ (d) absorption from the same spectrum.

Figure 5 shows a typical IRRA spectrum of the MMM monolayer after injection of the AH peptide. The vibrational bands observed in this spectrum show that both, lipids and peptides are present at the interface. The CH_2_ symmetric and anti-symmetric stretching vibrations (labeled a) originate from the lipid acyl chains. Their positions (υ_as_(CH_2_) = 2924.5 cm^−1^ and υ_s_(CH_2_) = 2854.5 cm^−1^) are indicative of acyl chains with a high content of *gauche* conformers, i.e., for lipid monolayers in the liquid expanded (LE) state [49,50,51]. Therefore, we conclude that the experimental conditions are appropriate to represent the fluid state of biological membranes. Furthermore, the carbonyl stretching vibrational band (labeled c) originates from vibrations of the lipid ester groups. The presence of the amide I’ band (labeled d) shows that the AH peptide indeed adsorbed to the monolayer interface. On a H_2_O subphase, the amide I band would be superposed by the OH bending vibration (1600 cm^−1^–1750 cm^−1^) of the subphase water. A rather small amide I signal of a short and only weakly adsorbed peptide such as AH would not be observable. All IRRAS measurements were therefore performed on a D_2_O subphase, which absorbs at lower wavenumbers (OD bending: 1150 cm^−1^–1300 cm^−1^) and thereby does not interfere with the amide I’ vibrational band. (The ’ indicates that acidic hydrogens of the amide bond are exchanged by deuterium.)

The position of the amide I’ band is sensitive to the secondary structure of a peptide, because it is influenced by the strength and geometry of the hydrogen bonds as well as by transition dipole moment coupling within ordered structures [36,52]. The amide I’ band of the AH peptide in its deuterated form is centered at 1645 cm^−1^. This position can be attributed to either an α-helix or a random coil conformation [36,37], which cannot be unambiguously distinguished on the basis of only one IRRA spectrum. However, analysis of a whole set of spectra, recorded at different AoI and polarization can help to differentiate between these two possibilities.

**Figure 6:**
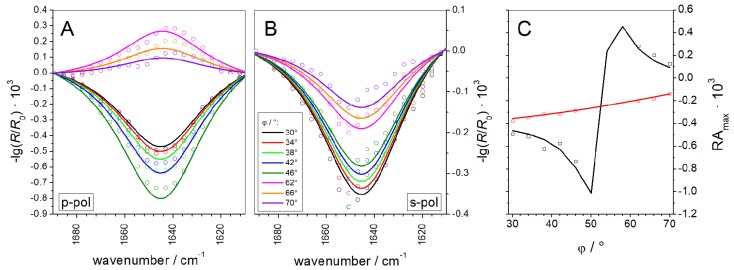
Experimental (lines) and best fitting simulated (dotted) IRRA spectra in the amide I region of the AH peptide adsorbed to an MMM monolayer in: p-polarization (**A**) l and s-polarization (**B**), and at different AoI, as indicated in the figure. (**C**) Intensities of experimental (symbols) and simulated (lines) amide I bands at their extrema. Black: p-polarization and red: s-polarization.

Starting after 7 h of equilibration (see bar in Figure 5) we recorded IRRA spectra at AoI between 30° and 70° in increments of 4°. At each angular step one spectrum in p-polarization and one in s-polarization were acquired. The long equilibration time was necessary: (i) to allow the film to relax; and (ii) to ensure a stable water and D_2_O vapor atmosphere within the IR light path. To further minimize spectral interferences with water rotation–vibrational bands as well as to improve the signal-to-noise (S/N) ratio, several cycles of angle and polarization dependent spectra were recorded and averaged. The resulting set of baseline corrected spectra is presented in Figure 6A,B (solid lines). Even though the bands are of low intensity and poor S/N ratio, the experimental spectra could be fitted by simulated ones using the optical model of Kuzmin and Michailov [30,31]. In our fitting procedure, we did not only rely on the fitting of the maximal or integral intensities of the amide bands, an approach that might introduce uncertainties due to the low S/N ratio. Rather, we fitted the complete band contour in the range of 1630 cm^−1^–1660 cm^−1^. Spectra recorded with an AoI close to the Brewster angle and in p-polarization were excluded from the fit, due to the very low reflectivity under these conditions. In principle, IRRAS probes the orientation of the transition dipole moment (TDM) of a certain vibration with respect to the electrical field vector of the incoming IR light. To calculate the tilt angle *θ* of the AH peptide from the band intensities the angle between the TDM and the helix main axis has to be known. This angle *α* has been determined by several groups and several approaches using different model helices [38,53,54,55], resulting in a variety of reported *α* values, ranging from 34°–42°. In our fitting procedure, *α* was set to a constant value, whereas *θ* was a free fitting parameter. As *α* and *θ* are interdependent, fits of equal goodness were achieved with input values for *α* between 34° and 40°, leading to helix tilt angles *θ* in the range of 72°–84°. For *α* > 40° the goodness of fitting decreased, indicating that these values are too high for our system. A very thorough analysis by Marsh et al. [53] gave a value of *α* = 38°, which is in our opinion the most reliable value. The fit of the experimental data with *α* = 38° is presented in Figure 6 (dotted lines) and results in a tilt angle of *θ* = 77°, with respect to the surface normal. To facilitate visual assessment of the fit quality Figure 6C shows the maximal band intensities of experimental and simulated bands as a function of the AoI. The determined tilt angle (*θ*) can also be expressed in terms of an order parameter

(4) S=12(3⋅cos2θ−1)=−0.42

The non-zero order parameter proves that the peptide does not adsorb in random coil conformation and eliminates the uncertainty about the secondary structure adopted by the AH peptide. If a random coil conformation can be excluded, the amide I’ band position unambiguously shows that the peptide is helical.

**Figure 7:**
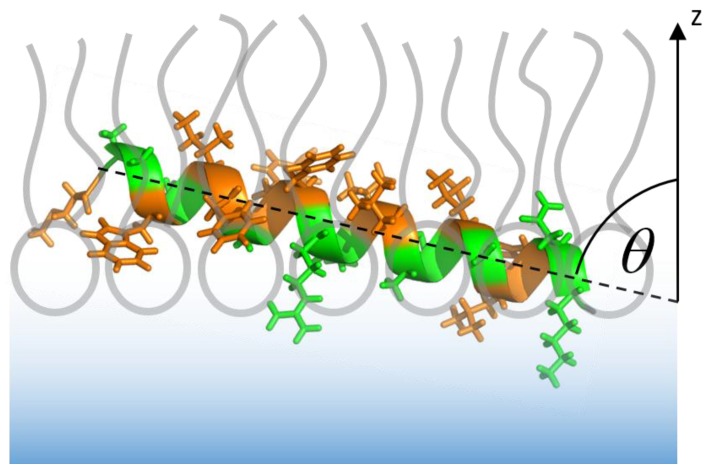
Orientation of the AH after insertion into an MMM lipid monolayer. The angle *θ* between helix axis (dotted) and monolayer normal (*z*-axis) is 77°, as determined by AoI and polarization dependent IRRA spectroscopy. The depth and the direction of insertion are proposed based on the distribution of hydrophobic (orange) and hydrophilic (green) residues and are not an experimental outcome of the IRRAS measurement. Peptide and lipid monolayer are drawn schematically and to scale.

Interestingly, the AH peptide does not insert completely parallel to the interface of the lipid monolayer. The IRRAS band fitting suggests that the helix inserts slightly tilted into the monolayer, as depicted in Figure 7. This might be due to the unequal distribution of hydrophobic and hydrophilic residues along the helix axis. Indeed, the C-terminus is a slightly more hydrophilic than the N-terminus, which might lead to a higher affinity of the C-terminus for the aqueous subphase or the polar lipid headgroup region, as opposed to a higher affinity of the N-terminus for the hydrophobic acyl chain region. The insertion of the N-terminal half of the helix into the membrane is 2.9 kJ/mol more favorable than the insertion of the C-terminal half, when partition free energies are calculated according to the Wimley and White octanol scale [41] (or 3.8 kJ/mol based on the interfacial scales [46]). This slight misbalance might lead to the inclined insertion of the N-terminal AH of Sar1 into the lipid membrane. In addition, the positively charged amino acids are located at the C-terminal end of the AH, which may lead to specific interactions of the C-terminal end with negative lipid head group charges. The finding is reasonable from a biological point of view: Since the C-terminus of the AH peptide is where the AH is attached to the water soluble Sar1 protein, it cannot be buried in the membrane. The slightly deeper insertion of the N-terminus into the membrane leaflet may serve to convey a stable anchorage.

### 3.2. The Complete Sar1 Protein

#### 3.2.1. Sar1 Adsorption at the Air–Water Interface

**Figure 8:**
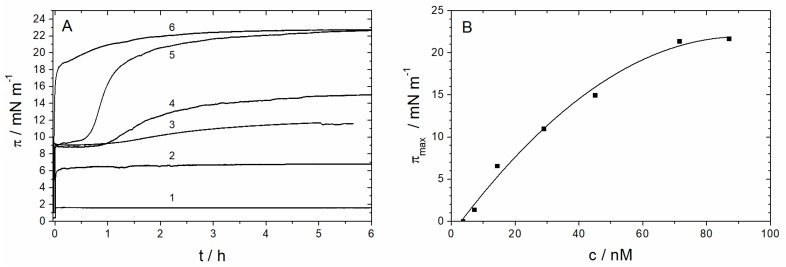
(**A**) Surface pressure versus time for the adsorption of Sar1 to the air–water interface at different protein concentrations ((1) 7 nM; (2) 15 nM; (3) 30 nM; (4) 45 nM; (5) 70 nM; and (6) 90 nM) starting with the injection of the appropriate volume of the Sar1 stock solution into the subphase. (**B**) Maximal surface pressure π_max_ after Sar1 adsorption as a function of the Sar1 concentration in the trough (T = 22 °C, HKM buffer in H_2_O). The value π_max_ was determined 5 h after Sar1 injection (from panel A).

Before studying the incorporation of the complete Sar1 protein (including the N-terminal, AH-forming amino acids) into lipid monolayers, we first investigated the adsorption behavior of the protein to the air/water interface (Figure 8). For final protein concentrations in the trough between 15 and 90 nM, the surface pressure increased immediately after injection of the protein and GTP into the subphase, indicating that the protein is surface active (Figure 8A). At protein concentrations of 30 nM and higher, the surface pressure increases rapidly, achieving a first plateau less than 1 h. Then, π further increases until it reaches a second plateau within several hours. At higher protein concentrations (70 nM and 90 nM), the surface pressure increases up to final value of 23 mN/m.

Figure 8B shows the maximum surface pressure as a function of the total protein concentration in the subphase. The maximum value of π approaches 23 mN/m at a protein concentration around 80 nM. Sar1 is therefore as surface-active as the N-Ras protein [20], which also belongs to the Ras superfamily of membrane-associating GTPases.

The structure and orientation of Sar1 at the air–water interface is unknown. Since the adsorption experiments were performed in the presence of GTP, the GTPase is probably in its activated conformation with the AH exposed. However, it remains unclear if the AH directs the protein into a preferred orientation at the interface.

#### 3.2.2. Sar1 Adsorption at the Lipid Monolayer

**Figure 9:**
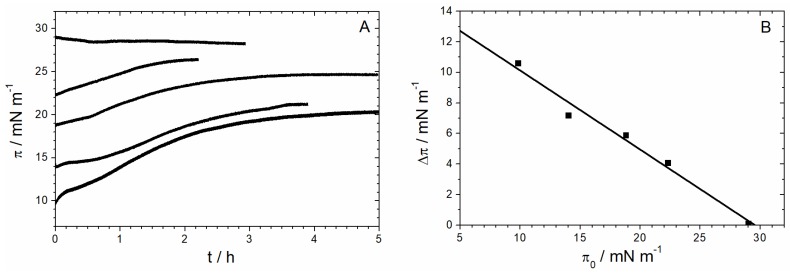
(**A**) Surface pressure versus time for the insertion of Sar1 into MMM monolayers at different starting pressures (π_0_) and a Sar1 subphase concentration of 10 nM. (**B**) Increase in surface pressure ∆π after insertion of Sar1 as a function of the initial surface pressure π_0_ of an MMM-monolayer (10 nM protein, HKM buffer, at 22 °C). The value ∆π was determined 2 h to 5 h after Sar1 injection, when the surface pressure π has reached a plateau value (from panel A).

To study the ability of Sar1 to insert into a lipid monolayer, the MMM lipid mix was spread at the air–water interface up to various surface pressures between 10 and 30 mN/m. After a short relaxation period, Sar1 and GTP were injected into the subphase underneath the lipid monolayer. After several hours, the surface pressure reaches a maximum value (Figure 9A).

Plotting the measured increase in surface pressure ∆π against the initial surface pressure π_0_ usually leads to a straight line with a negative slope, which intersects the abscissa at the so-called maximum insertion pressure (MIP) [13,14,15]. It is generally assumed that a protein spontaneously inserts into membranes if this value is lower or equals the monolayer–bilayer equivalence pressure of about 30 mN/m [56,57]. When the initial surface pressure of the lipid monolayer is higher than the MIP, the protein does not spontaneously insert into the monolayer. Figure 9B shows that Sar1 has a maximum insertion pressure of 30 mN/m, which indicates that this protein spontaneously inserts into biological lipid membranes.

#### 3.2.3. IRRAS

**Figure 10:**
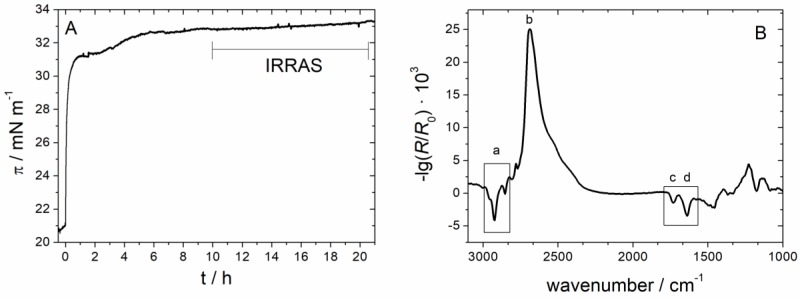
(**A**) Time course of surface pressure change during adsorption of Sar1 (100 nM in bulk) to an MMM monolayer at 21 mN/m. Sar1 and GTP were injected underneath the monolayer at t = 0 h. Angle dependent IRRA spectra were acquired between 10 h and 20 h after insertion, as indicated in the figure. (**B**) Average of IRRA spectra recorded in p-polarization and at an AoI of 42°. For band assignments, see Figure 5.

To gather more detailed, spectroscopic information about the interaction of Sar1 with the lipid monolayer we performed IRRAS measurements in a similar manner as reported above for the isolated AH peptide. Again, the experiment was performed on a D_2_O buffer to avoid a superposition of amide I and water absorption bands. Spectra were recorded after an extended equilibration time to minimize water and D_2_O vapor contributions to the spectra. The time dependent Sar1 adsorption, which was further studied by IRRAS, is depicted in Figure 10A. The MMM lipids were spread up to a surface pressure of about 21 mN/m, a value below the proposed monolayer–bilayer equivalence pressure of 30 mN/m. At this pressure, the monolayer density is only about 15% lower than in a typical bilayer (see MMM isotherm in Figure 4). This slightly lower lipid density was chosen to obtain a sufficiently high amount of protein inserted into the monolayer for IRRAS detection, which is only possible at initial surface pressures below the MIP (see Section 3 and Figure 9). The lower initial pressure and lipid packing density is necessary because, in contrast to biological membrane bilayers, the monolayer is not allowed to expand upon insertion of the protein. The final surface pressure after insertion of the protein is 32 mN/m (Figure 10A), i.e., a pressure at which the lipid packing is comparable to that of biological systems. The injected Sar1 concentration is 100 nM, which is the same as the AH peptide concentration in the experiment described above. Interestingly, the pressure increase directly after injection is also similar for the two experiments. This implies that Sar1 only inserts with its N-terminal AH into the lipid monolayer, assuming that the adsorbed amounts in the two experiments are comparable. After equilibration, the Sar1 containing monolayer is more stable and the ∆π values are higher as compared to the monolayer containing only the AH peptide. This may be due to either an additional insertion of other parts of the Sar1 protein or to the adsorption of a higher amount of protein compared to the AH peptide.

A typical IRRA spectrum, recorded 10 h after injection of Sar1 underneath an MMM monolayer is presented in Figure 10B. The same spectral features are observable as discussed above for the adsorption of the isolated AH peptide (Figure 5). The CH_2_ stretching vibrations indicate that the monolayer is in a LE state and the presence of an amide I’ band shows that Sar1 has adsorbed to the MMM monolayer. However, some differences can be recognized in comparison with the spectrum of the AH peptide: firstly, the OD stretching vibrational band centered at 2600 cm^−1^ is more intense. The intensity of this band increases with layer thickness [34]. Therefore, it can be concluded that the MMM/Sar1 monolayer is thicker than the MMM/AH monolayer. As the thickness of the MMM monolayer is expected to be the same in both cases, the higher total layer thickness can be attributed to the adsorption of the bulkier Sar1 protein. Based on the conclusion made above that only the N-terminal AH is inserted into the lipid monolayer, the major part of Sar1 will be located at the monolayer interface, causing an increase in layer thickness. The intensity of the OD stretching vibrational band therefore lends additional support to the conclusion that Sar1 binds the membrane by inserting only the AH. Secondly, the intensity ratio of the amide I’ band (d) to the lipids’ carbonyl band (c) and is much higher in the case of the MMM/Sar1 spectra. This is due to the much higher number of amino acid (aa) excited by the IR light in case of Sar1 adsorption (192 aa) compared to AH peptide adsorption (23 aa). It shows that the protein is sensed by the IR light even though it is not inserted into but located underneath the lipid monolayer. The higher band intensity of the amide I’ band also leads to a better S/N ratio, which is a prerequisite for performing the data analysis described below. Thirdly, the center frequency of the amide I’ band is lower than 1645 cm^−1^, the band is wider, and consists of multiple components. This is due to the different secondary structure elements of the complete protein, which contribute to the spectrum. Besides α-helices, now also 3_10_-helices, β sheets, turns and loops as well as some unstructured regions contribute to the over-all amide I’ band, all absorbing at slightly different wavelengths and with different intensities.

**Figure 11:**
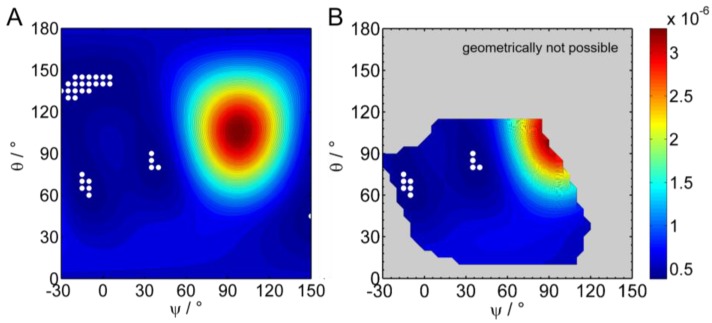
(**A**) Sum square deviation (SSD) of simulated IRRA spectra of Sar1 fitted to experimental ones at various protein orientations, defined by tilt angle *θ* and twist angle *ψ*. The experiments were performed in the presence of GTP. Blue color denotes a good spectral fit and therefore a probable protein orientation; red colored regions denote a bad spectral fit at the tested protein orientations und therefore unlikely protein orientations. All points within a 95% confidence interval around the minima of this error map are marked with white symbols. (**B**) The same plot as in panel A, but only the SSD values of geometrically possible orientations are shown. All Sar1 orientations that are geometrically not possible because the N-terminus is too far away from the monolayer have been masked out. Orientations are classified as possible, when the N-terminal attachment point of the AH (Gly24) is within a distance of 10 Å from the membrane interface. The color bar represents the color codes for SSD values and applies to both panels.

To gather orientational information on the complete Sar1 protein, IRRA spectra under variation of AoI and polarization were recorded for the MMM/Sar1 monolayer. However, due to the complexity of the amide I’ band, the data evaluation is much more challenging than for the isolated helix. No precedent can be found in the literature, where the orientation of a complete protein was determined by IRRAS. In one study, the amide I’ band contours of the N-Ras protein were analyzed by qualitative comparison of experimental spectra with simulated ones in selected orientations [20], but the simulation did not yet account for all protein features and did not aim to reproduce the experimental spectra. In another study, AoI and polarization dependence of a complex band consisting of 3 subcomponents was fitted to determine an orientation of a liquid crystal at the air/water interface [34]. However, the analysis of protein amide I’ bands is much more complex, because many more components contribute to this band. Recently, we used advanced IRRAS amide I’ band analysis to reveal the reorientation of a protein domain (helical domain of the dynamin EDH2) upon lipid membrane binding [58]. The developed method has been adopted here and further improved to determine the orientation of Sar1 in its membrane bound state. Sar1 consists of 6 β-sheets and 8 helices, each oriented differently and thus contributing with a different AoI and polarization dependence to the amide I’ contour. Additionally, turns, unordered regions and the differences in parallel and antiparallel β-sheets as well as the difference between α- and 3_10_ helices have to be taken into account. In total, 24 subcomponents of the amide I’ band were defined, which are described by 16 parameters. A set of spectra in the amide I’ band region was recorded using 16–18 different conditions, i.e., AoI and polarization of the IR light. We developed a method to analyze this complex set of amide I’ bands and were able to retrieve information about the protein orientation. Details of the method are described in the Materials and Methods (Section 2.2.5). Briefly, the internal orientations of all secondary structure elements of the protein are determined from its crystal structure [2] (pdb 1M2O, see Figure 2) and their contributions to the amide I’ contour at every AoI and polarization is calculated. Then, the three-dimensional protein model is rotated in a systematic manner about tilt angles *θ* and twist angles *ψ* (for definitions, see Supplementary Materials, Figure S4) and the amide I’ contour is reevaluated. All calculated sets of amide I’ bands are compared with the experimental one to determine the protein tilt and twist angles that lead to the best band fit. The sum square deviation (*SSD_θ,ψ_*) between simulated and experimental bands, i.e., the goodness of the band fit, is represented in a color coded error map (error hypersurface) as a function of protein tilt and twist angle (Figure 11A). In this representation, blue areas denote a good and red areas denote a bad fit between simulation and experiment, with the best possible parameter set at the given protein orientation. Based on this analysis of the IRRAS spectra, a huge range of protein orientations can clearly be excluded. Conversely, the map has three minima at orientations described by (*θ*, *ψ*) = (70°, −10°), (*θ*, *ψ*) = (85°, 35°), and (*θ*, *ψ*) = (140°, −15°). At and around these points the tilt and twist angles describe with a high probability the orientation of the membrane bound Sar1. Spectroscopically, it cannot be distinguished which of these orientations reflects the true Sar1 orientation(s).

However, in the case of Sar1, the results of the spectroscopic analysis can be combined with geometrical considerations. As discussed above, the protein binds by insertion of its N-terminal AH. The protein must therefore be oriented in a way that the N-terminus of the truncated protein structure determined by crystallography (Gly24) points towards the membrane interface. Thus, all the Sar1 orientations where the Gly24 is too far away from the interface can be excluded. We therefore calculated the distance of Gly24 at every possible rotation of Sar1 (Supplementary Materials, Figure S5) and classified all orientations where Gly24 is located within 10 Å from the surface as possible orientations, all others as “geometrically not possible”. An overlay of this geometrical classification and the spectroscopic evaluation is shown in Figure 11B. Here, the error of amide I band fitting is only evaluated in the range of geometrically possible orientations. All other orientations are shown in gray. As a result, some orientations that yielded a good fit spectroscopically can be excluded by geometrical considerations, in particular the minimum around (*θ*, *ψ*) = (140°, −15°). Vice versa, the range of geometrically possible orientations is further confined by the spectroscopic analysis, as can be seen from the red areas in the range of geometrically possible orientations.

This shows that the orientation of Sar1 is not solely determined by the insertion of the AH. The vicinity of the N-terminal amino acids is a necessary but not sufficient criterion to determine the Sar1 orientation in the membrane bound state. Other factors must be constraining the orientational freedom of the protein. These may include specific or electrostatic interactions of amino acids at the membrane binding interface with the lipid headgroups or further geometrical constraints.

**Figure 12:**
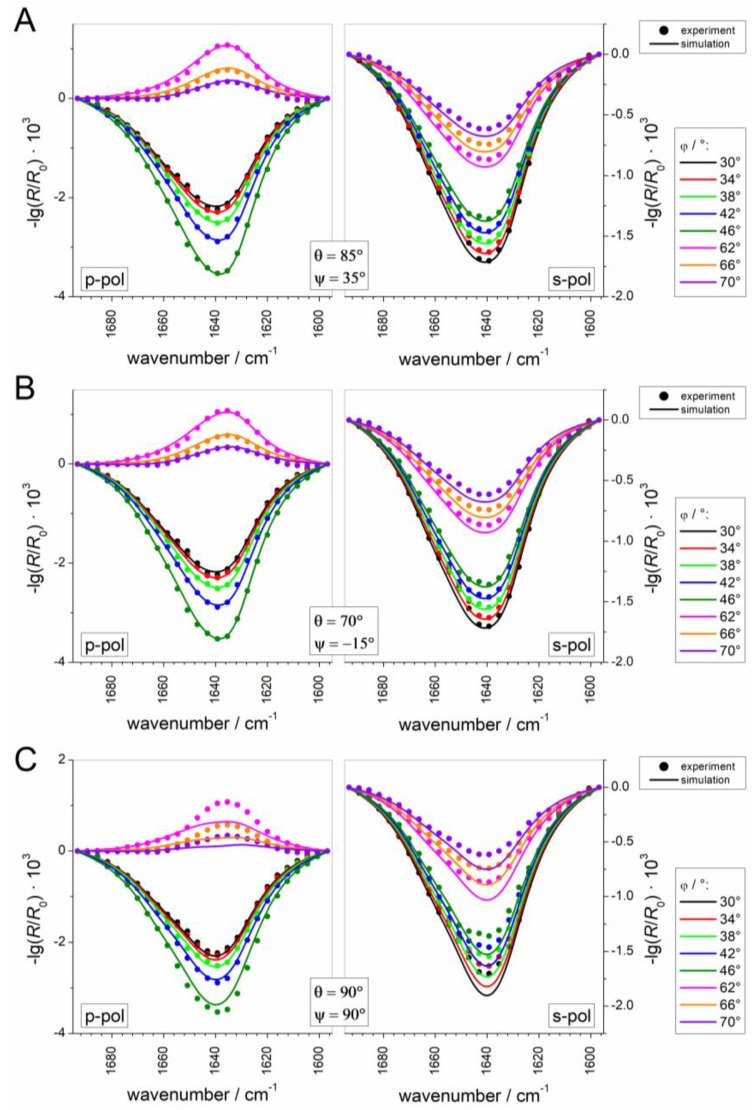
Experimental spectra (symbols) of Sar1 adsorbed to an MMM monolayer in the presence of GTP, recorded at various angle of incidence *φ* and polarizations (p or s). Spectra are shown along with the best fitting simulated spectra (lines) for different protein orientations: (**A**,**B**) probable protein orientations, i.e., at minima in the error map shown in Figure 11; and (**C**) a protein orientation that is classified as geometrically possible but results in a bad fit to the spectra, close to the red-colored maximum in the error map in Figure 11A,B.

To get a visual impression of the quality and deviations of the fits in probable (blue regions) and improbable orientations (red regions), the experimental IRRA spectra are presented together with the best fitting simulated ones at three given protein orientations in Figure 12: at the two minima of the error map ((*θ*, *ψ*) = (85°, 35°) (A) and (*θ*, *ψ*) = (70°, −15°) (B)) and in an orientation that is classified as geometrically possible, but yields a bad spectroscopic fit ((*θ*, *ψ*) = (90°, 90°) (C)). Comparison of the three sets of spectra shows that the amide band shape is highly sensitive to the orientation of the protein and demonstrates the power of IRRAS as a method for determining protein orientations.

**Figure 13:**
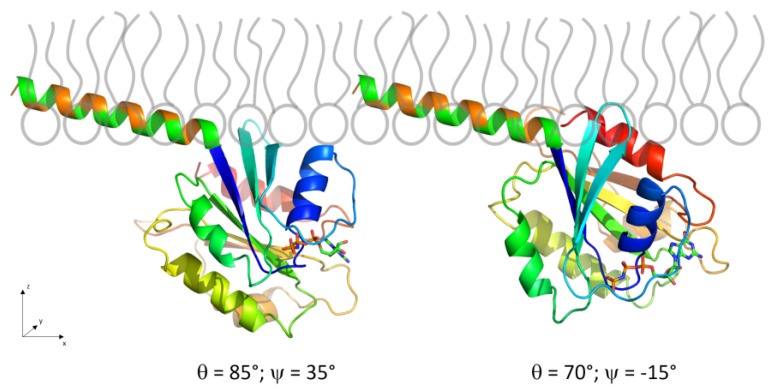
Most probable orientations of Sar1 with respect to the lipid monolayer as determined from angle dependent IRRA spectroscopy. These two orientations correspond to the minima of the error map shown in Figure 11B. The AH is attached to the model in the orientation determined above. It was present in the experiment and included in the simulation in this fixed orientation and not rotated about *θ* and *ψ* as the rest of the model. The lipid monolayer is shown schematically and in scale to the protein model.

In Figure 13, Sar1 is depicted in its most probable orientations after adsorption to an MMM monolayer, as determined by angle and polarization dependent IRRAS. The N-terminal AH is not shown in the figures, because it is not part of the crystal structure, which was the basis for the calculations. Nevertheless, it was present in the experiment and included in the simulations in the orientation that was determined before (always tilted by 77° with respect to the layer normal, independently of the protein tilt and twist angles). In both orientations shown in Figure 13, the N-terminus of the crystal structure, i.e., the point of attachment of the AH, faces the monolayer. Two more observations of biological interest can be made: (i) The nucleotide binding site is accessible for Sec12, which binds Sar1 and promotes the nucleotide exchange, before Sar1 exposes its AH and binds to the membrane [59]. (ii) The binding site on Sar1 for Sec23/Sec24 (the COPII component that is recruited by membrane-bound Sar1) is also accessible when Sar1 is bound to the membrane in one of these orientations.

Besides AH, some other amino acids may interact with the lipids in both calculated orientations. In the orientation shown on the left hand side of Figure 13, this is the loop between sheets 2 and 3 and parts of the corresponding strands (turquoise; residues 61–69, ELAIGNIKF) as well as the C-terminal end of helix 1 (blue), which contains Lys44. Especially the two lysines might contribute to Sar1 binding by electrostatic interactions with negatively charged lipid headgroups. In the solution given on the right hand side of Figure 13 the same loop between sheet 2 and 3 may interact with the membrane, as well as the C-terminal helix (red; residues 181–190: EAFQWLSQY). The latter, however, does not seem to be a typical membrane binding motive.

The presented orientations correspond to the two minima of the error map that was calculated with a given set of fixed parameters (for definitions and methods of evaluation, see Materials and Methods and Supplementary Materials) and a set of free fitting parameters that were varied during a fit at every probed tilt/twist combination. Slight variations in the starting parameters and spectral pretreatment yield slight variations in the error map. Therefore, orientation with somewhat different tilt and twist angles (roughly ±10°) are also possible and still yield a reasonably good spectral fit. Probably, Sar1 has some degree of orientational freedom, by virtue of its anchorage to the lipid monolayer by the N-terminal AH. A more rigid confinement might be achieved after binding of the other COPII coat proteins, Sec23/34 and Sec13/31 (see below).

#### 3.2.4. Time Dependency/Stability

The data were recorded over a time span of 10 h (10 to 20 h after injection, see Figure 10A). During this time, four cycles of spectra recordings were performed, i.e., at each condition (AoI, polarization) four spectra were recorded and averaged with a time difference of 2.5 h between the single spectra. We can therefore address the question whether during this time the amount of bound Sar1 and its orientation remains stable. As Sar1 is a GTPase, the hydrolysis of GTP may influence the binding state over this long period, even though an excess of GTP was injected together with the protein. Indeed, we observed a decrease in integral band intensity over time, which could in principle stem from a loss in bound protein or a protein reorientation (Supplementary Materials, Figure S6). To distinguish between these possibilities, we analyzed the spectra of each cycle of recordings separately, without averaging over time. As the amount of bound protein is a free fitting parameter during the analysis, this yields a temporal sequence of four error maps for orientation prediction and four associated measures for the protein concentration. The results are provided in the Supplementary Materials (Figure S7). They show that the orientation of bound Sar1 remains overall unaffected but the concentration of bound protein slightly decreases over time. To clarify if GTP hydrolysis is responsible, and to circumvent this problem, the Sar1 adsorption and IRRAS experiment and analysis were repeated with the non-hydrolysable GTP analogue GMP-PNP.

#### 3.2.5. Adsorption and Orientation of Sar1 in the Presence of GMP-PNP

**Figure 14:**
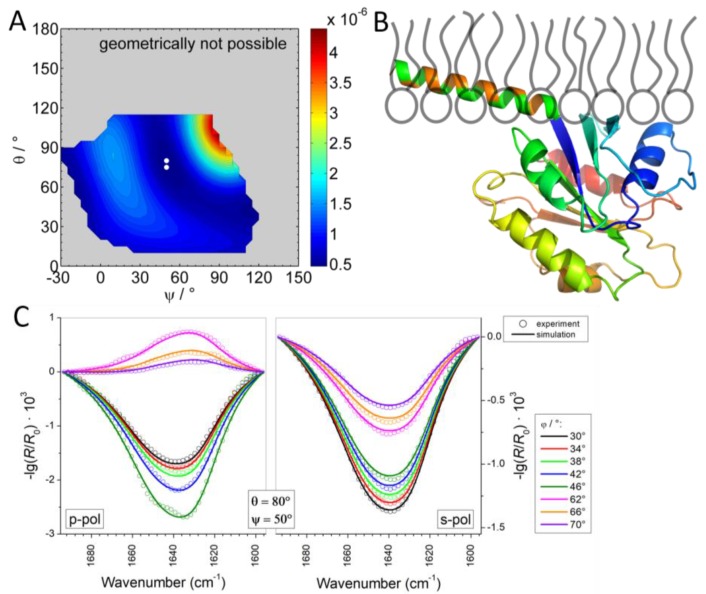
Analysis of the orientation of Sar1 (100 nM) adsorbed to an MMM monolayer in the presence of GMP-PNP (2 µM). (**A**) Map of sum square deviation (SSD) between experimental spectra and best fitting simulated ones as a function of Sar1 orientation (*θ*, *ψ*). The white symbols mark data points within the 95% confidence interval of an F statistic around the minima of the hypersurface. The *SSD*_φ,ψ_ is only shown for geometrically possible orientations. Geometrically not possible orientations are masked out by gray color. The color bar represents the color codes for SSD values. (**B**) Representation of Sar1 in the orientation described by the minimum of the SSD hyperplane (*θ* = 80°, *ψ* = 50°). The AH is attached to the model in the orientation determined above. It is included in the simulation without being rotated about *θ* and *ψ* as the rest of the model. (**C**) Experimental (symbols) and simulated (lines) spectra at the minimum of the SSD hypersurface.

The adsorption isotherm of Sar1 to an MMM monolayer in the presence of GMP-PNP is shown in the Supplementary Materials (Figure S8). Experimental conditions were the same as in the above described experiments. The pressure increase after Sar1/GMPNP injection is also comparable to the increase in case of Sar1/GTP adsorption, indicating that a similar amount of protein adsorbed to the monolayer and that GMP-PNP functionally replaces GTP as expected [1,8]. IRRA spectra were now recorded in eight cycles until 38 h after injection of Sar1/GMP-PNP. The surface pressure (Supplementary Materials, Figure S8) as well as the IRRA amide band intensity (Supplementary Materials, Figure S9) remained constant over the complete measurement time. This indicates that Sar1 remains indeed stably adsorbed to the MMM lipids when nucleotide hydrolysis is impeded. The result of the orientation analysis of the time averaged data is shown in Figure 14. The best spectral fits were achieved for a Sar1 orientation defined by a tilt angle *θ* = 80° and a twist angle *ψ* = 50°, with respect to the crystal structure (see Figure 14A,C). In this orientation the N-terminus of the Sar1 crystal structure is oriented towards the interface, allowing the insertion of the AH into the monolayer or membrane. It is also similar to one of the two likely orientations determined for GTP-loaded Sar1 ((*θ*, *ψ*) = (85°, 35°), Figure 13, left side). In this orientation, the protein might also interact with the membrane with parts of β-strands 2 and 3 as well as the loop separating the two strands (residues 60–70: EELAIGNIKFT) which are facing the lipid layer. Especially Lys68 might assist the binding by electrostatic interaction with the lipid headgroups. In addition, Lys44 (C-terminal end of helix 1) might be involved in the interaction (Figure 14B).

#### 3.2.6. Comparison to Sar1 Orientation after Sec23/24p and Sec13/31p Recruitment

**Figure 15:**
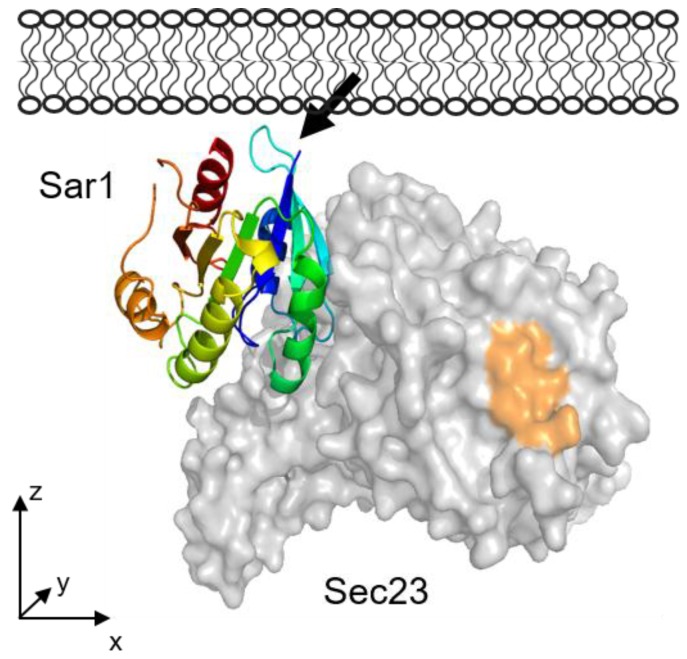
Orientation of Sar1/Sec23 within the complete COPII coat bound to a lipid membrane, as determined by cryo electron tomography [60]. The rest of the COPII coat proteins (Sec24, Sec13 and Sec31) are not depicted in this scheme. The binding site for Sec 24p is indicated in orange. The bold arrow indicates Gly24, i.e., the point of attachment of the AH membrane anchor.

The structure of the complete COPII coat on a lipid bilayer was previously investigated by cryo electron tomography [60]. Zanetti et al. fitted crystal structures of the various COPII subunits as rigid bodies into an electron density obtained from subtomogram averaging, and obtained a model of the complete COPII coat, including Sar1, bound to a lipid bilayer. Figure 15 shows the orientation of Sar1 and Sec23 as part of the COPII coat under GMP-PNP conditions [60]. Applying the computational rotation scheme from this work, the orientation of Sar1 in the cryo-EM complex structure corresponds to a tilt of *θ* = 80° and a twist of *ψ =* −2° of the Sar1 pdb structure 1M2O. Hence, the tilt is similar, but the twist is lower in comparison with Sar1 alone as determined by IRRAS in the presence of GMP-PNP ((*θ*, *ψ*) = 80°, 50°). The difference in orientations can be directly seen by comparing Figure 15 and Figure 14B. It suggests a slight reorientation of Sar1 during the binding of the further COPII components.

Visual comparison of the Sar1 orientation from the cryo-EM complex ((*θ*, *ψ*) = (80°, −2°),Figure 15) with the two most probable orientations of Sar1 alone activated by GTP ((*θ*, *ψ*) = (85°, 35°)) and ((*θ*, *ψ*) = (70°, −15°)), Figure 13) suggests that the Sar1 orientation within the complex is close to one of these orientations ((*θ*, *ψ*) = (70°, −15°)) and lies within a range of possible orientations of membrane-bound Sar1.

It is reasonable to think that the experimentally determined, average orientations differ from one step to the next in COPII coat assembly. Here, we investigated the first step of coat assembly, i.e., the binding of Sar1 prior to recruitment of further coat components. Zanetti et al. determined the orientation of Sar1 within the complete COPII coat. The recruitment of Sec23/24 and Sec13/31 may select a preferred orientation from an ensemble of Sar1 orientations, leading to the change in the experimentally determined average values.

## 4. Conclusions

In the present study, we show that the protein Sar1 stably binds to monolayers whose composition was previously optimized for reconstitution experiments of the COPII complex [1]. By monolayer adsorption experiments coupled with infrared reflection-absorption spectroscopy (IRRAS) several aspects of binding were investigated.

It is known that the N-terminal stretch (residues 1–23) of Sar1 is essential for membrane binding [2,40]. Here, we show that this stretch forms an amphipathic helix (AH) in contact with lipids and inserts into the lipid monolayer. Interestingly, the N-terminal peptide does not adsorb to a bare air/water interface, i.e., it is not surface active. Apparently, the lipids play a major role in the formation of the amphipathic structure. Probably, only the specific hydrophobic, hydrophilic and electrostatic interactions of the lipid acyl chains and head groups with amino acids of different philicities along the peptide stretch lead to the formation of the amphipathic helix structure. Although helix induction upon membrane binding was found for several peptides [61,62] and intrinsically disordered proteins [63,64], no such report exists for the N-terminal region of Sar1.

We could further show that a lipid monolayer is sufficient for AH insertion, which suggests that the insertion of the Sar1 AH is not transmembrane. This conclusion is confirmed and refined by the determination of the AH insertion angle by means of angle and polarization variation IRRA spectroscopy. This method revealed that the AH inserts nearly parallel to the plane of the monolayer, with a slight inclination of 13° (77° with respect to the monolayer normal). This insertion geometry corresponds very well to the radial and axial distribution of hydrophobic and hydrophilic amino acids in the AH. It allows the contact of the more hydrophobic amino acids with the acyl chain region and the exposure of the more hydrophilic amino acids to the polar and more hydrated lipid headgroup region. The observed inclination of the AH also allows the globular GTPase domain of Sar1 to reside in the aqueous subphase, while the other, N-terminal end of the AH extends into the hydrophobic acyl chain layer. This organization supports the current picture of Sar1 biological function. The insertion of the AH into only the proximal leaflet of the ER membrane is expected to generate membrane curvature [65,66], which is a prerequisite for transport vesicle formation [8]. The location of the globular domain in the aqueous phase allows recruitment of the other COPII components.

Studying the lateral surface pressure upon injection of Sar1 protein into the subphase of MMM monolayers, we found that, similar to the isolated N-terminal Sar1 peptide, the full-length protein is able to insert into the lipid layer. The maximum insertion pressure (MIP) is determined to be 30 mN/m. This implies that the full-length Sar1 spontaneously inserts into lipid bilayers, since this value corresponds to the monolayer–bilayer equivalence pressure at which the packing density of lipids within a monolayer is comparable to bilayer densities [56,57].

From the fact that the pressure increase upon insertion of only the N-terminal peptide and the pressure increase upon insertion of the complete protein are comparable, we conclude that only the AH of Sar1 inserts into the monolayer and the remaining protein body is located in the aqueous subphase (representing the cytosol in the cell). The protein binds in the presence of GTP as well as in the presence of its non-hydrolysable analogue GMP-PNP. It is known from previous studies that GTP or a GTP analog is required to release the N-terminal stretch from a binding pocket within the globular domain of Sar1 [2,40]. It is expected that in the GMP-PNP-bound state, Sar1 will be permanently bound to the membrane while, in the GTP-bound state, the nucleotide may slowly hydrolyze, causing the AH to retreat to the binding pocket, leading to the dissociation of Sar1 from the membrane. The protein concentration and orientation in the monolayer bound state remains constant for long periods (up to 40 h) when GMP-PNP is present. Conversely, the concentration of bound Sar1 decreases in the presence of GTP. We conclude that the hydrolysis of GTP leads to desorption of the protein from the lipid layer. In the future, this hypothesis could be further tested by varying the GTP concentration in the experiment, controlling the hydrolysis conditions, and monitoring the hydrolysis state. Furthermore, binding experiments on bilayers [67] could be used to compare the long-term stability of Sar1 binding under GTP-hydrolyzing and non-hydrolyzing conditions.

Development of a method based on IRRA spectroscopy in combination with the available crystal structure of Sar1 [2], (RCSB entry 1M2O) has led to a prediction of the orientation of monolayer-bound Sar1. We could show that not every orientation that allows insertion of the AH into the monolayer is adopted by the protein with the same probability. Rather, the spectroscopic results substantially refine the conceivable range of protein orientations. This observation indicates that, besides the insertion of the AH, additional interactions between the protein surface and the lipid layer influence the orientation of the bound Sar1.

The determined orientation is compared to the orientation of Sar1 within the complete COPII coat formed on a lipid bilayer, which was determined by cryo-electron tomography [60]. The orientations determined by the two independent methods and in two different environments are similar. However, the recruitment of the other COPII components and the concomitant coat polymerization seem to lead to a slight readjustment or preferential selection of a certain Sar1 orientation.

This study demonstrates how monolayer experiments in conjunction with IRRAS can be used to elucidate details of the interaction of amphiphilic polymeric molecules with lipid assemblies. An advantage of the monolayer setup is that experimental parameters can be easily controlled and adjusted. In particular, they can be chosen to match biological conditions. The defined orientation of the lipid layer in this experimental setup enables an advancement of spectroscopic methods that reveal the orientation of adsorbed or inserted molecules or molecular moieties. The experimental method and data analysis that has been introduced in this study of the Sar1-membrane interaction can be used and adopted to study the adsorption and orientation of other membrane interacting or surface active molecules. With this study, we aim to contribute both to a deeper understanding of Sar1 membrane binding as an initial step in COPII transport vesicle formation and to the advancement of a method for studying the orientation of molecules at interfaces.

## Supplementary Materials

The following are available online at www.mdpi.com/2073-4360/9/11/612/s1, Figure S1: Sar1 second derivative ATR spectrum, Figure S2: Determination of band parameters, Table S1: Band parameters, Figure S3: Determination of layer thickness and refractive index, Table S2: Secondary structure elements of Sar1, Figure S4: Definition of rotation angles, Figure S5: Geometrically allowed orientations, Figure S6: Time dependent intensity decrease for Sar1/GTP, Figure S7: Temporal stability of membrane bound Sar1, Figure S8: Time-course of adsorption of Sar1 in the presence of PNP-PNP, Figure S9: Time dependent intensity for Sar1/GMP-PNP, Figure S10: Sar1/GMP-PNP orientation.
