# Supplementary material for: Binding of the GTPase Sar1 to a Lipid Membrane Monolayer: Insertion and Orientation Studied by Infrared Reflection–Absorption Spectroscopy
Source: Polymers (Basel). 2017 Nov 14;9(11):612. doi: 10.3390/polym9110612 (PMC6418733; doi:10.3390/polym9110612)
Supplement: Supplementary file 1 [file polymers-09-00612-s001.pdf]

Supplementary Materials: Binding of the GTPase Sar1 to a Lipid Membrane Monolayer: Insertion and Orientation studied by Infrared Reflection-Absorption Spectroscopy

Christian Schwieger *, Annette Meister, Sebastian Daum, Alfred Blume, Kirsten Bacia *

1. Start parameters for band positions, $\upsilon_{0(1\text{-}5)}$

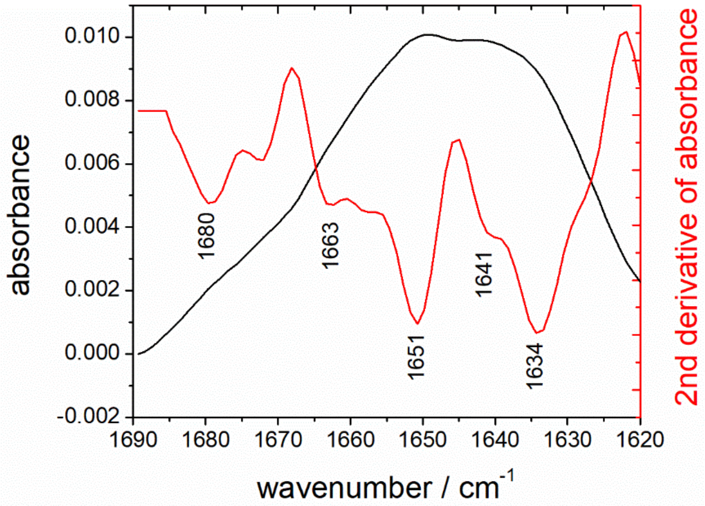

Figure S1. Determination of the position of amide I′ subcomponents by ATR-IR spectroscopy of Sar1. Amide I′ band (black) and its second derivative (red) are shown to reveal the underlying subcomponents. The positions of these components (black numbers in the figure) serve as starting parameters for the further analysis. For the measurement a Sar1 stock solution (prepared in HKM buffer) was freeze dried and rehydrated in D_2O based HKM buffer. A Buffer/D_2O spectrum was subtracted before further analysis. The measurement was performed on a BIO-ATRII cell (Bruker) at 20 °C.

2. Refinement of band positions (υ_0) and full width at half heights (*fwhh*) by fit of IRRAS bands with monolayer model

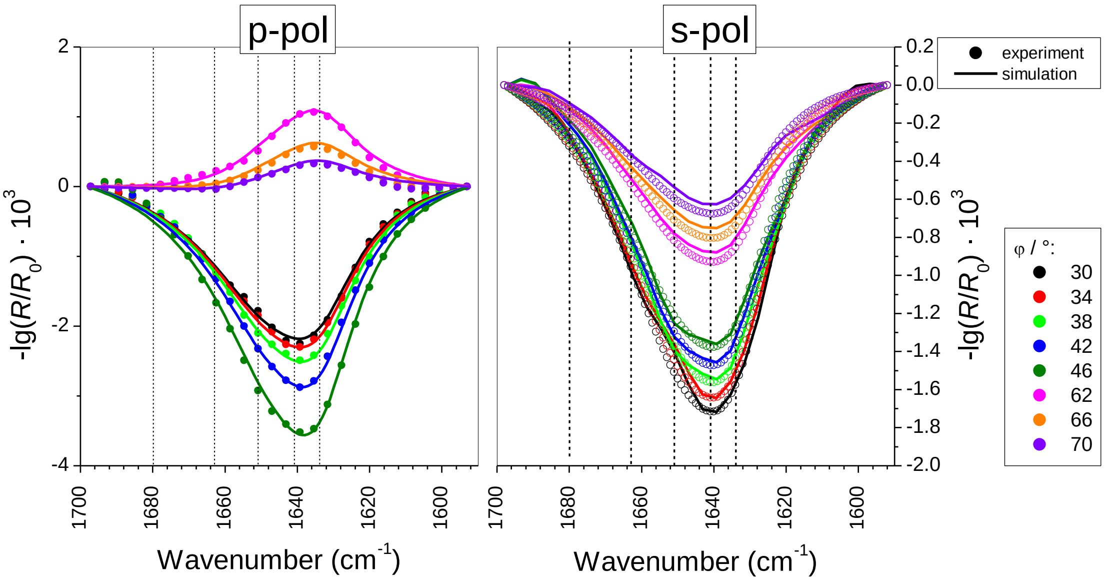

Figure S2. Determination of band parameters. Sets of experimental spectra (symbols) of Sar1

adsorbed to a MMM lipid monolayer spread on an HKM buffer prepared in D_2O and best fitting simulated spectra (lines). Shown is the spectral region of the amide I′ band after correction for water vapor contribution (experimental spectra) and linear baseline subtraction in the presented range (experimental and simulated spectra). The spectra are recorded with IR light in p-polarization (left panel) and s-polarization (right panel) and at different incidence angles ϕ (see legend). The simulated spectra contain 5 subcomponents for β-sheet, unordered stretches, α-helices, turns and a high wavenumber component for antiparallel β-sheets (from right to left). The positions of the subcomponents are indicated by the vertical dotted lines.

Table S1. Band parameters determined from the fit of IRRA spectra shown in Figure S2

Secondary structure element	ν_0 / cm^{-1}	*fwhh* / cm^{-1}
β-sheet	1634	27
unordered stretches	1637	37
helix	1647	36
turns	1660.5	31
antiparallel β-sheet	1682	49

3. Determination of the layer thickness and refractive index of the interfacial layer

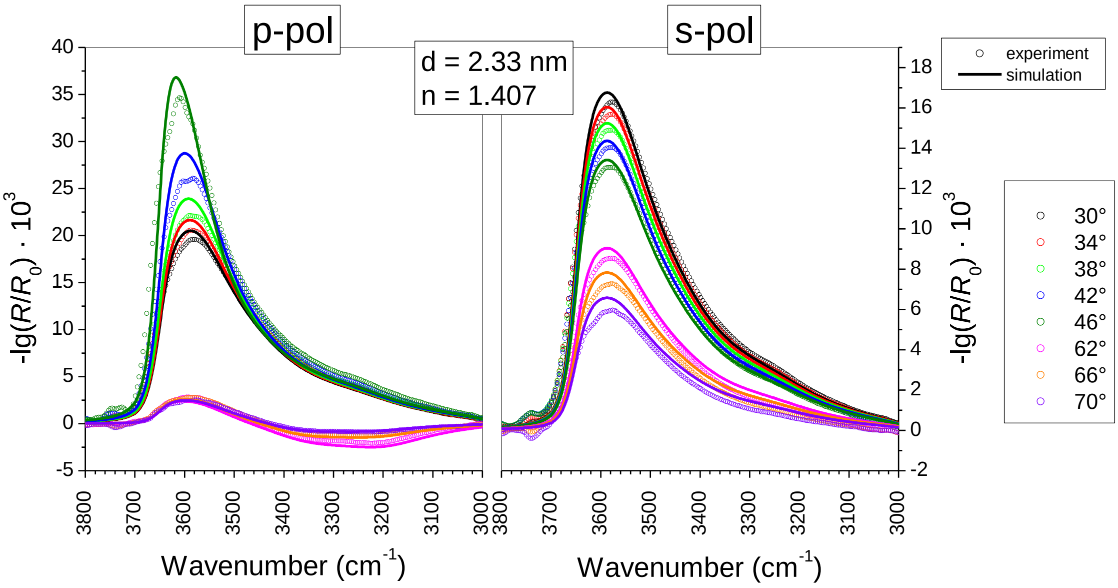

Figure S3. Determination of layer thickness and refractive index. Sets of experimental (symbols) and simulated (lines) IRRA spectra of Sar1 adsorbed to a MMM lipid monolayer spread on an HKM buffer prepared in H_2O. Shown is the spectral region of the OH stretching vibration. The spectra are recorded with IR light in p-polarization (left panel) and s-polarization (right panel) and at different incidence angles ϕ (see legend). The best fit of the simulated spectra to the experimental spectra was achieved using a layer thickness d = 2.33 nm and a refractive index of the interfacial film (includes lipids and protein) of n = 1.407.

4. Assignment of secondary structure elements

Table S2. Secondary structure elements of Sar1 as in pdb 1M2O and its N-terminal amphipathic helix (AH). The type of sheets can be parallel (p) or antiparallel (ap). Sheets that appear as one sheet in the structure representation may be divided into subsheets for the analysis in order to be able to assign different major and minor axes, which is done to account for different twist angles in strongly twisted sheets.

structure element	type	residue numbers
helix 1	α	35–46
helix 2	3_{10}	77–81
helix 3	3_{10}	83–88
helix 4	3_{10}	102–104
helix 5	α	105–118
helix 6	3_{10}	119–123
helix 7	α	141–149
helix 8	α	178–188
AH	α	1–23 (+2)[a]

[a] The full length protein contains 2 additional amino acids (Gly, Ser) at the N-terminus, due to the protein production and purification protocol.

structure element	number of residues
unordered	33
turns	32

structure element	type	residue numbers
sheet 1a	p	58–62
sheet 1b	p	63
sheet 2a	ap	67–72
sheet 2b	ap	73
sheet 3	p	25–30
sheet 4a	p	93–96
sheet 4b	p	97–99
sheet 5a	p	127–128
sheet 5b	p	129–132
sheet 6a	p	166–169
sheet 6b	p	170

5. Definition of the coordinate system and rotation angles

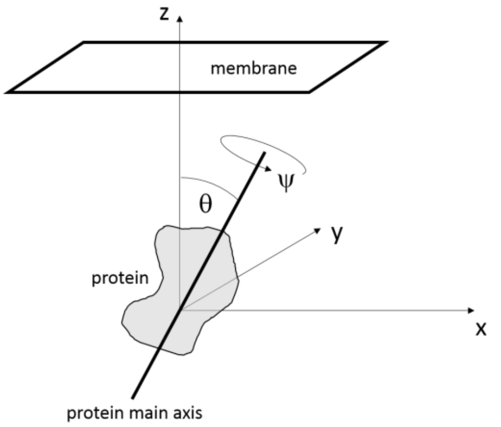

Figure S4. Lab coordinate system with definitions of the tilt angle θ, the twist angle ψ, and the position of the membrane parallel to the x-y plane and on the positive z-axis with respect to the protein.

6. Method to determine allowed rotations

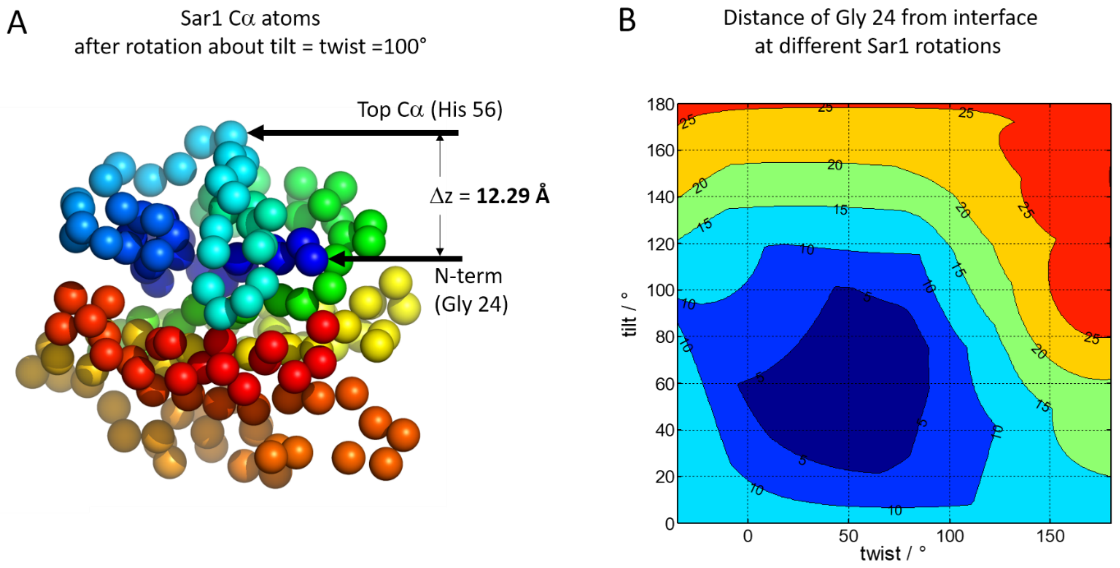

Figure S5. Geometrically allowed orientations. A: Illustration of the distance calculation for the example of tilt = 100° and twist = 100°. Gly24 is the first resolved amino acid in the X-ray crystal structure (1M2O). The interface is presumed to be in the x-y plane and at the z-position of the uppermost Cα atom of Sar1. For the distance calculation, the z-positions of the respective Cα atoms were evaluated. For the final analysis we assumed all orientations with Δz ≤ 10 Å to be allowed orientations (dark blue and blue areas in B). The orientation shown in A would be classified as "not possible". B: Distance of Gly24 from the interface for all possible combinations of tilt and twist angle, i.e. protein orientations.

7. Time dependence / stability of monolayer-bound Sar1p in the presence of GTP

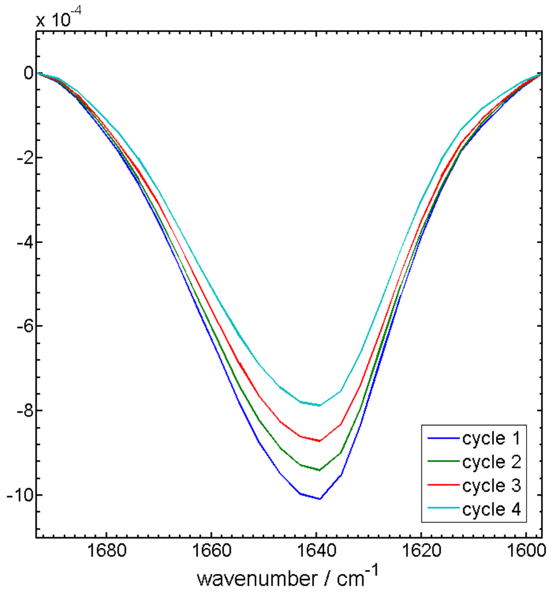

Figure S6. Time-dependent intensity decrease. Average of all spectra of Sar1 in the presence of GTP recorded in s-polarization from 4 consecutive cycles of spectra recording. Each cycle of recording takes approximately 3 h. The region of the amide I′ vibration is shown. Note that the intensity of the amide I′ band decreases with time.

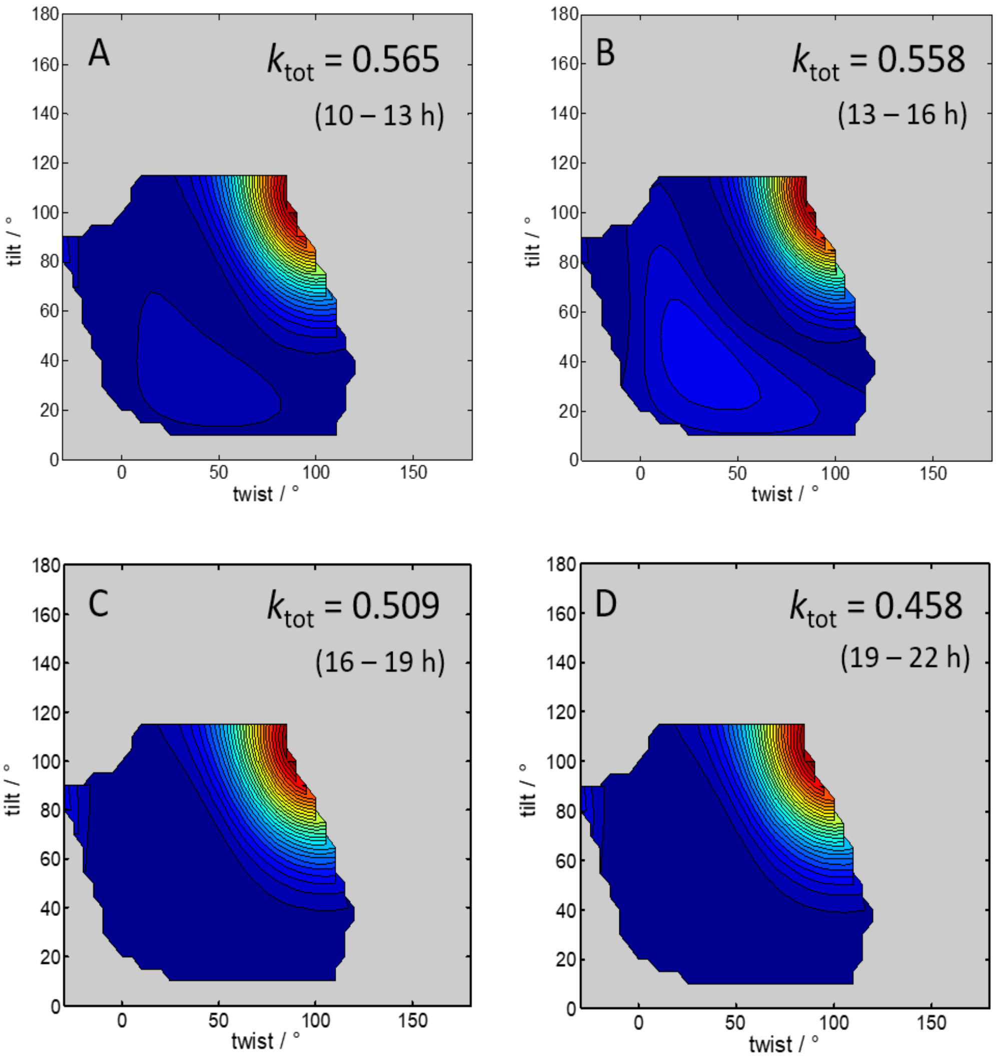

Figure S7. Temporal stability of membrane bound Sar1. Analysis of single cycles of IRRAS measurement (variation of AoI and polarization), without averaging over time. Maps of sum square deviations (*SSD*) between simulated and experimental spectra recorded at **A**) 10–13 h, **B**) 13–16 h, **C**) 16–19 h, and **D**) 19–22 h after injection of Sar1 and GTP (16 fold excess) below an MMM monolayer. Blue color denotes a good spectral fit; red color denotes a bad spectral fit. The grey area denotes orientations that are geometrically not possible. The value of the total absorption coefficient, k_{tot}, is a measure for the protein concentration at the interface. k_{tot} is a free fitting parameter in the analysis. The values given in the panels are determined from the best spectral fit at the minimum of the error hyperplane. Note that the orientations denoted by the minima of the error planes do not change much over time, whereas k_{tot} systematically decreases with time.

8. Adsorption of Sar1 to MMM in the presence of GMP-PNP

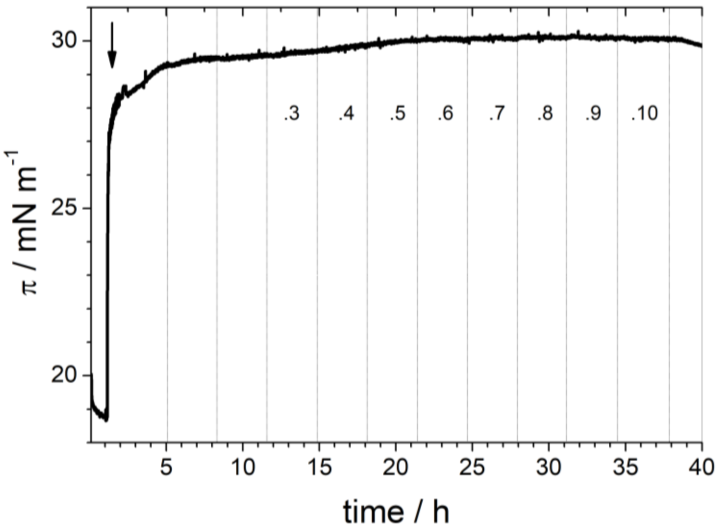

Figure S8. Time-course of adsorption of Sar1 (100 nM) to an MMM monolayer spread on a D_2O / HKM buffer subphase in the presence of GMP-PNP (2 μM). The numbers in the figure denote the number of the cycle of the IRRA spectra recordings that were included in the analysis.

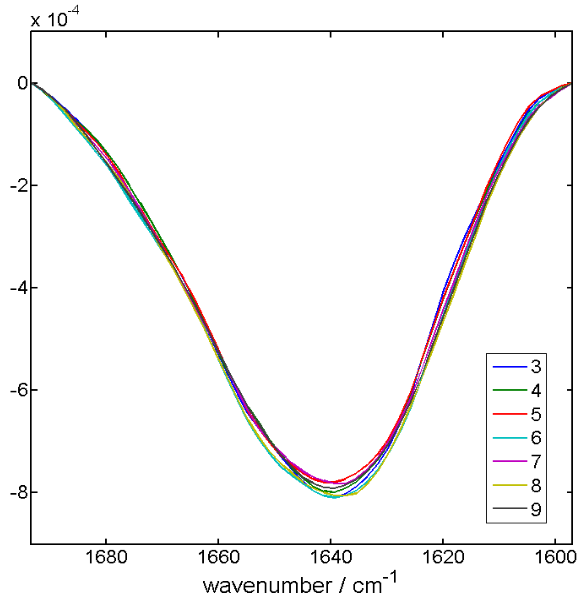

Figure S9. Time dependence of the intensity. Average of all spectra of Sar1 in the presence of GMP-PNP recorded in s-polarization of all consecutive cycles of spectra recording, included in the orientation analysis. Each cycle of recording takes approximately 3 h. The region of the amide I′ vibration is shown. Note that the intensity of amide I′ band is stable over time with GMP-PNP as opposed to GTP (Fig. S6).

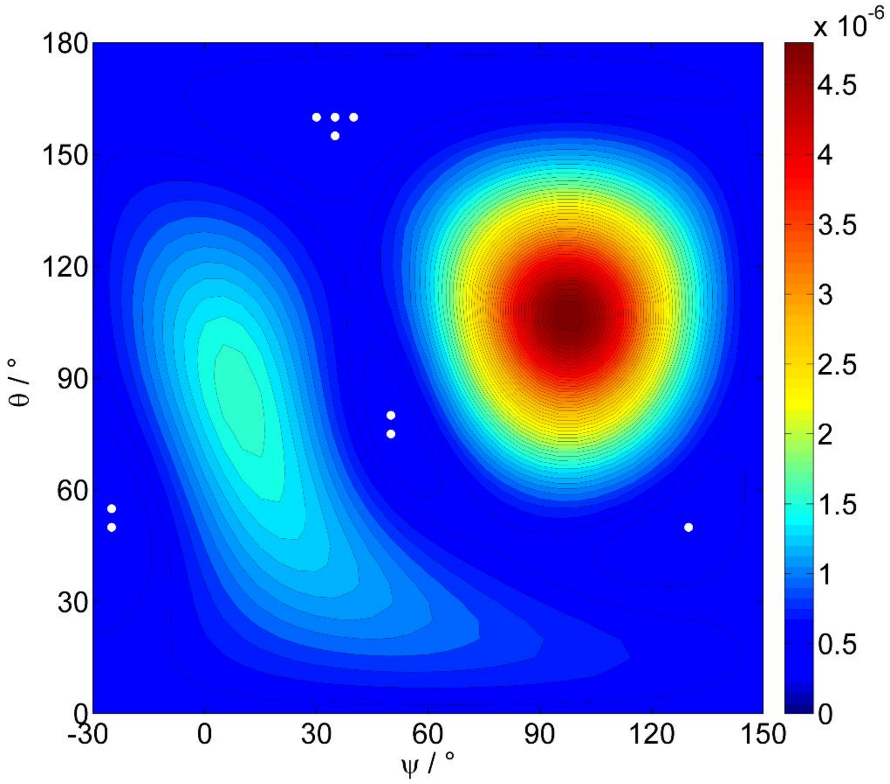

Figure S10. Sar1/GMP-PNP orientation. Map of sum square deviation (*SSD*) of best fitting simulated spectra to experimental spectra of Sar1 in the presence of GMP-PNP. Blue color denotes a good spectral fit and a probable protein orientation; red color denotes a bad spectral fit and an improbable protein orientation. The white symbols mark the data within a 95% confidence interval of an F value statistic test.
